# Retracing the origin and evolution of a cryptic antimicrobial peptide within mammalian lactoferrin

**DOI:** 10.1371/journal.pbio.3003932

**Published:** 2026-08-25

**Authors:** Titas Sil, Caitlin H. Kowalski, Sierra Scamfer, Natalie Copeland, Matthew F. Barber

**Affiliations:** 1 Institute of Ecology and Evolution, University of Oregon, Eugene, Oregon, United States of America; 2 Department of Biology, University of Oregon, Eugene, Oregon, United States of America; 3 Department of Dermatology, Dartmouth Hitchcock Medical Center, Lebanon, New Hampshire, United States of America; 4 Institute of Molecular Biology, University of Oregon, Eugene, Oregon, United States of America; Fred Hutchinson Cancer Research Center, UNITED STATES OF AMERICA

## Abstract

Antimicrobial peptides (AMPs) constitute key components of innate immunity across the tree of life. Canonical AMPs are typically translated as small proteins and secreted from host cells to act against microbes. However, cryptic AMP-like domains are also embedded within diverse proteins not classically associated with antimicrobial function. How such embedded AMPs first emerge and diversify remains unclear. Here we retrace the origin and evolution of the abundant mammalian protein lactoferrin and its embedded AMP, lactoferricin. By resurrecting extinct lactoferrin ancestors dating back to the earliest mammals, we identify an enrichment of cationic and hydrophobic amino acids in the lactoferricin domain over time. These changes enabled ancient lactoferricin to first rupture bacterial membranes, an activity that was later enhanced in extant mammals conferring potent bactericidal activity. In addition, we find that natural selection within the lactoferricin domain has continued to modulate antimicrobial activity on recent evolutionary timescales. In particular, we pinpoint a single rapidly evolving site in lactoferricin among great apes that significantly enhances antimicrobial potency against major pathogenic bacteria. Together, our study illustrates how novel immune protein functions can arise, evolve, and diversify to strengthen host defense against microbial pathogens.

## Abstract 2

How antimicrobial peptides embedded within larger proteins arise remains unclear. This study resurrects ancient proteins to reveal how antimicrobial peptides emerged and diversified in mammalian lactoferrin.

## Introduction

The evolution of immune function is vital for species survival in response to pathogen antagonism [1–3]. Antimicrobial peptides (AMPs), also known as host defense peptides, are major contributors to innate immunity across all domains of life, displaying a range of antimicrobial and immunomodulatory activities [4–7]. Well-characterized AMPs are typically small (less than 50 amino acids) and positively charged, promoting interactions with negatively charged microbial cell envelopes [4,5,8]. These interactions can lead to membrane disruption, cell lysis, and impairment of essential cellular processes, ultimately inhibiting microbial growth or triggering cell death [8]. Beyond canonical AMPs, an increasing number of studies have identified the presence of AMP-like domains embedded within larger proteins [9–13]. These domains can in some cases be liberated through proteolytic cleavage, suggesting a mechanism by which diverse proteins may acquire immune functionality. At present, little is known regarding the origins of these embedded AMP domains or the molecular changes that enabled the acquisition of antimicrobial activities.

**Fig 1:**
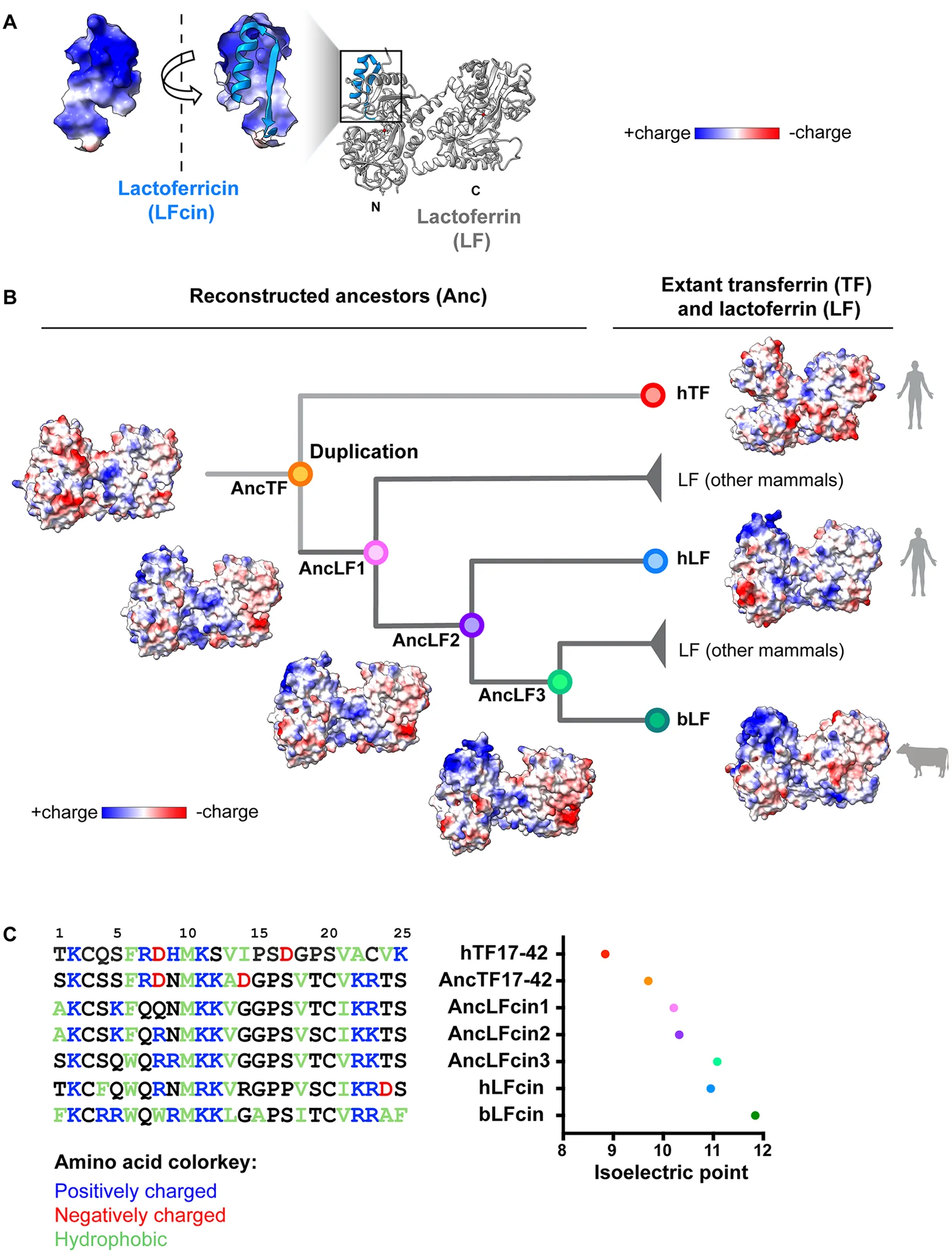
Reconstructing the emergence of antimicrobial function in mammalian lactoferrin. **(A)** Structure of the N-terminal lactoferricin region of lactoferrin (PDB: 1lfg), enriched with positively charged amino acids. **(B)** Phylogenetic tree of reconstructed ancestral lactoferrin homologs (AncTF and AncLF) with extant human transferrin (hTF, PDB: 3qyt), human lactoferrin (hLF, PDB: 1lfg), and bovine lactoferrin (bLF, PDB: 1blf) illustrated with surface electrostatic potentials. Structures of ancestral proteins were predicted using AlphaFold2. AncTF represents the last common ancestor of mammalian transferrin and lactoferrin, after which the first lactoferrin ancestor (AncLF1) arose by gene duplication. AncLF2 is the common ancestor of hLF and bLF, while AncLF3 represents a bLF ancestor. **(C)** Alignment of the lactoferricin region from hTF, AncTF, selected AncLFs, hLF, and bLF, with their corresponding isoelectric points. Higher isoelectric points indicate a higher incidence of positive charges. Dot colors in the isoelectric point follow node colors in the phylogenetic tree (Fig 1B), corresponding to the respective proteins. The data underlying this Figure can be found in Table A of S1, S2 and S3 Data.

The mammalian protein lactoferrin provides an exemplar for the evolution of antimicrobial functions. Lactoferrin is an abundant secreted iron-binding protein that arose by duplication of the transferrin gene in the ancestor of placental mammals ~160 million years ago (S1A Fig) [14,15]. Transferrin proteins are typically composed of two homologous domains, termed the N- and C-lobes, each of which binds a single ferric iron ion with high affinity [15–18]. Serum transferrin mediates iron-transport in the bloodstream, delivering this essential metal nutrient to cells via receptor-mediated endocytosis [15]. Transferrin family proteins can also provide a defensive benefit by sequestering iron from invasive pathogens, a process termed “nutritional immunity” [19–21]. Lactoferrin, in contrast to transferrin, is abundantly expressed in mammalian milk, colostrum, tears, secondary granules of neutrophils, and other body fluids [22,23]. Lactoferrin also possesses unique antimicrobial functions which are absent in extant transferrin (S1A–S1B Fig) [23]. For example, the N-terminal region of the lactoferrin N-lobe can be proteolytically cleaved by abundant host proteases such as pepsin, trypsin, and chymotrypsin to generate cationic AMPs, including lactoferricin and lactoferrampin [24–29]. These peptides are capable of killing diverse bacterial and fungal pathogens as well as inhibiting microbial biofilm formation [26,29–32]. Lactoferrin-derived AMPs share key features with conventional AMPs, including a high proportion of positively charged and hydrophobic amino acids that confer amphipathic properties (Figs 1A, S2A). Despite its functional importance, the evolutionary origin of antimicrobial activity within the lactoferricin domain remains poorly understood. Previously, we found that lactoferricin exhibits evidence of repeated positive selection in simian primates, with codons possessing an elevated rate of nonsynonymous to synonymous substitutions (dN/dS) [33]. This suggests that lactoferricin has continued to diversify within mammals since the acquisition of antimicrobial activity. Determining how this novel function arose and subsequently evolved in an otherwise well-conserved iron-transport protein could offer broader insights into the origin of antimicrobial proteins and the mechanisms by which new biological functions arise. Here we retrace the molecular emergence of lactoferricin antimicrobial activity in ancestral mammals, as well as demonstrate how recent natural selection has enhanced the function of this abundant host defense protein.

## Results

### Ancestral lactoferrin accumulated positive charges during mammalian evolution

To investigate the origin and evolutionary history of the lactoferricin domain, we first compared lactoferricin sequences across diverse mammals (S2A Fig). Given that lactoferricin peptides have been reported of different lengths in different species, we focused our studies on the core 25 amino acids conserved across lactoferricin orthologs (amino acids 17–42 in mature human lactoferrin) [24,25]. Most homologs possessed elevated isoelectric points indicating enrichment of positively charged amino acids such as arginine (R), lysine (K), and histidine (H). They also contained interspersed hydrophobic residues characteristic of other AMPs. Despite these conserved physiochemical properties, lactoferricin amino acid sequences were highly diverse across orthologs, making it difficult to pinpoint amino acids that may have been responsible for antimicrobial function (S2A Fig).

To retrace the mutations that led to the emergence of a novel antimicrobial function, we reconstructed ancestral lactoferrin and transferrin sequences across diverse mammals (Fig 1B). Ancestral sequence reconstruction (ASR) provides a powerful approach to characterize the function of ancient proteins [34–36]. Using the Topiary software pipeline [37] and publicly available transferrin and mammalian lactoferrin sequences from NCBI, we predicted ancestral amino acid sequences of transferrin and lactoferrin proteins (S2B Fig). We focused on ancestral proteins (prefixed “Anc”) at key nodes of the phylogenetic tree where the lactoferricin domain underwent significant divergence (Fig 1B, 1C). Each predicted sequence was associated with posterior probability (PP) scores, which identified the most likely residue at each site as well as the most probable alternative (S3 Fig). These data were used to generate corresponding alternative (Altall_Anc) peptide sequences, in which alternative amino acid states with a PP greater than 0.25 replaced the most probable ancestral amino acid. To assess the robustness of our findings, we included both the primary ancestral reconstructions and their alternate versions in subsequent functional analyses (S4 Fig).

To compare the antimicrobial function of lactoferricin between ancestral and extant variants, we chose human and bovine lactoferrin (hLF and bLF, respectively) as extant references, given the extensive previous work on these orthologs in the field [22,24,25,27]. As a negative control we selected human serum transferrin (hTF) as it did not exhibit antimicrobial function in the N-terminal region (S1B Fig). We referred to the last common ancestor of mammalian transferrin prior to the gene duplication event as AncTF. Gene duplication produced the first ancestral lactoferrin, which we designated AncLF1 (Fig 1B). We also considered two intermediate ancestors, denoted AncLF2 and AncLF3. AncLF2 represents the last common ancestor of hLF and bLF in the gene phylogeny, and AncLF3 represents a bovine-specific ancestor that accumulated multiple substitutions in the lactoferricin region relative to AncLF2 (Fig 1C). We then predicted the structures of these full-length ancestral proteins using AlphaFold2 (Fig 1B). When the surface electrostatic potentials were compared, we observed a progressive increase in positive charge density (blue surface) in the N-terminal region of lactoferrin following the duplication event (Fig 1B). This suggests that the emergence of positively charged surfaces in the N-terminus occurred around the origin of lactoferrin itself in placental mammals.

We next examined the lactoferricin domains of these reconstructed lactoferrin ancestors, which we refer to as AncLFcin1, AncLFcin2, and AncLFcin3 (Fig 1C). As the corresponding region in transferrin (residues 17–42) does not form a known antimicrobial peptide, we referred to these homologous sequences as AncTF17–42 in ancestral transferrin and hTF17–42 in human serum transferrin. We observed that cationic amino acids became more abundant in lactoferricin post-duplication, with the emergence of a K5 substitution in AncLFcin1, R8 in AncLFcin2, and R9 in AncLFcin3 (Fig 1C). To quantify this trend, we calculated peptide isoelectric points, which increased from AncTF17–42 to bLFcin (Fig 1C). Furthermore, we noted the emergence of hydrophobic residues adjacent to positively charged amino acids in the lactoferricin domain after duplication (e.g., alanine 1 in AncLFcin1 and AncLFcin2). Based on these observations, we hypothesized that the increase in positive charge and hydrophobicity enabled lactoferricin to acquire antimicrobial function by promoting interactions with bacterial cell envelopes.

### Early origin of antimicrobial activity in lactoferricin

**Fig 2:**
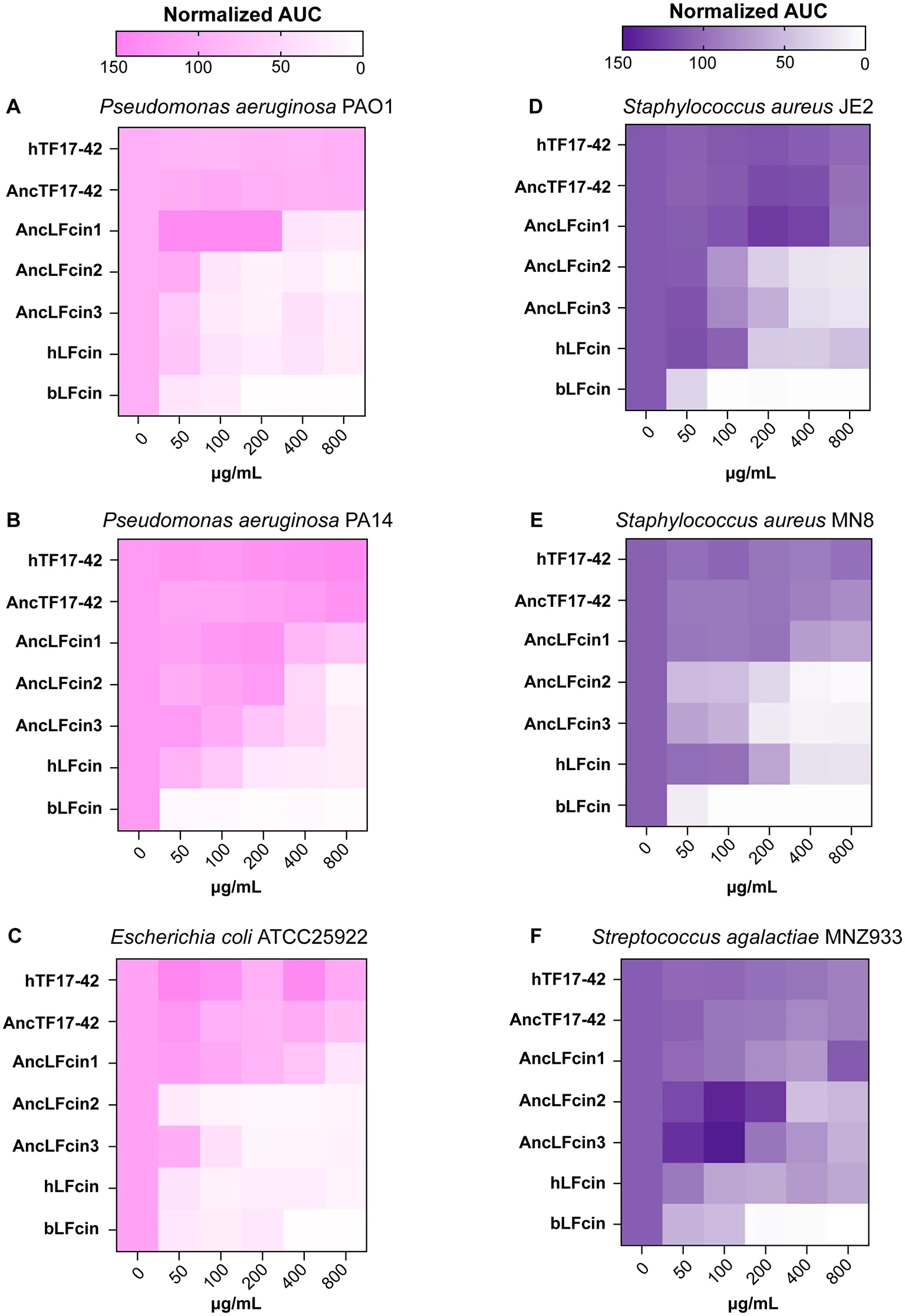
Early origin of antimicrobial activity in lactoferricin. Heatmaps depict area under the growth curves (AUC), normalized to the untreated control (100% = mean AUC of the no-peptide control; 0% = no growth), for peptides at the indicated concentrations. Color denotes normalized AUC (from 0% to 100%; darker = more bacterial growth). Gram-negative strains are shown in pink (*Pseudomonas aeruginosa* PAO1 and PA14, *Escherichia coli* ATCC 25,922; **A–C)**; Gram-positive strains in purple (*Staphylococcus aureus* JE2 and MN8, *Streptococcus agalactiae* MNZ933; **D–F)**. OD₆₀₀ values were blank-corrected by subtracting the OD₆₀₀ of cell-free wells containing an equivalent volume of medium with the matching peptide concentration (or no peptide) from that of the corresponding culture wells. For normalization of the AUCs in GraphPad, 100% was defined as the mean AUC of untreated bacterial cultures (no peptide) and 0% as an AUC of zero (no bacterial growth). Each cell represents the mean of three biological replicates. The data underlying this Figure can be found in Tables B–G of S1 Data.

To characterize the antimicrobial potency of ancestral and extant lactoferricin domains, we synthesized these peptides and tested their activity against several bacterial pathogens (Fig 2). Among gram-negative bacteria we included *Pseudomonas aeruginosa*, a pathogen frequently associated with nosocomial infections, chronic wounds, and cystic fibrosis, as well as a reference strain of the enteric bacterium *Escherichia coli* [38–40]. We also tested representative Gram-positive bacteria including *Staphylococcus aureus*, a major cause of invasive hospital and community-acquired infections, as well as *Streptococcus agalactiae*, a frequent agent of severe infections in neonates [41–43].

To compare the antimicrobial potency of ancestral and extant lactoferricin homologs, we measured bacterial growth in presence of peptides at various concentrations over 24 h. Human lactoferrin concentrations range widely in vivo from 7–8 mg/mL in colostrum, 1–3 mg/mL in breast milk, 0.1–2 mg/mL in tears and saliva, and even lower levels (0.001–0.1 mg/mL) in other secretory body fluids [44]. We therefore used physiologically relevant concentrations of each peptide, ranging from 0.1–1 mg/mL, to assess their dose-dependent antimicrobial activities. Bacterial growth at each concentration was measured by normalized area under the curve (AUC) calculations, where higher AUC corresponds to increased bacterial growth. Relative to the untreated control, peptides exhibiting <50% bacterial growth were classified as having high antimicrobial potency, those with 50%–75% growth as moderate, and those with >75% growth as weak. We observed that all bacteria grew to high levels in the presence of hTF17–42, indicating it has little to no antimicrobial activity as expected (Fig 2). Similarly, the ancestral-derived peptide AncTF17–42 exhibited negligible antimicrobial activity across all doses and pathogens (Fig 2). AncLFcin1, in contrast, reduced growth by >50% at higher doses against a subset of pathogens, for example at 400 µg/mL against *P. aeruginosa* PAO1 and at 800 µg/mL against *E. coli* (Fig 2A–2C). We noted that weakly active peptides, such as AncLFcin1, appeared to promote bacterial growth at low concentrations (Fig 2A). Such observations could reflect the ability of bacteria to use these peptides as a carbon source, or alternatively the emergence of resistant bacterial subpopulations. These findings suggest that antimicrobial activity in this domain arose around the time of the duplication event that gave rise to mammalian lactoferrin.

AncLFcin2, representing the last common ancestor of hLF and bLF, exhibited higher potency at lower concentrations across all pathogens tested relative to AncLFcin1 (Fig 2). For example, AncLFcin2 was highly potent at 100 µg/mL against *P. aeruginosa* PAO1, and at 50 µg/mL against *E. coli* and *S. aureus* MN8 (Fig 2A, 2C, and 2E). Against other pathogens, AncLFcin2 also reduced bacterial growth by >50% in the 100–400 µg/mL range. These results suggest that antimicrobial activity of the lactoferricin domain was substantially enhanced between AncLFcin1 and AncLFcin2. AncLFcin3 exhibited broad antimicrobial activity in the 100–800 µg/mL range but overall was less effective than bLFcin. To validate the robustness of our ASR, we also tested alternate versions of each ancestor against *P. aeruginosa* and *S. aureus* (S4 Fig). Overall trends in antimicrobial activity were largely consistent with those observed for the primary reconstructed ancestors. In general, gram-negative bacteria were found to be more susceptible to these peptides that Gram-positive strains, consistent with previous observations in the field regarding many cationic AMPs [5,45]. The only exception was Altall_AncTF17–42, which exhibited weak activity against *S. aureus* at the highest concentrations (S4 Fig).

Among extant lactoferricin orthologs, bLFcin was consistently more potent than hLFcin against all pathogens tested, significantly reducing bacterial growth at concentrations as low as 50 µg/mL (Fig 2). Notably, hLFcin was more effective against gram-negative bacteria where it restricted growth at 100–200 µg/mL. Against Gram-positive bacteria, the antimicrobial potency of hLFcin only became evident at concentrations above 200 µg/mL. We also observed that AncLFcin2 was more effective than hLFcin against Gram-positive bacteria. Overall, these data indicate that modest antimicrobial activity was present in AncLFcin1, around the emergence of lactoferrin in ancient mammals, which then increased in subsequent ancestors. However, given that hLFcin exhibited weaker antimicrobial activity than some earlier ancestors, our findings also suggest that the evolution of lactoferricin antimicrobial function did not increase uniformly during mammalian evolution.

### Enhancement of bactericidal activity during lactoferricin evolution

**Fig 3:**
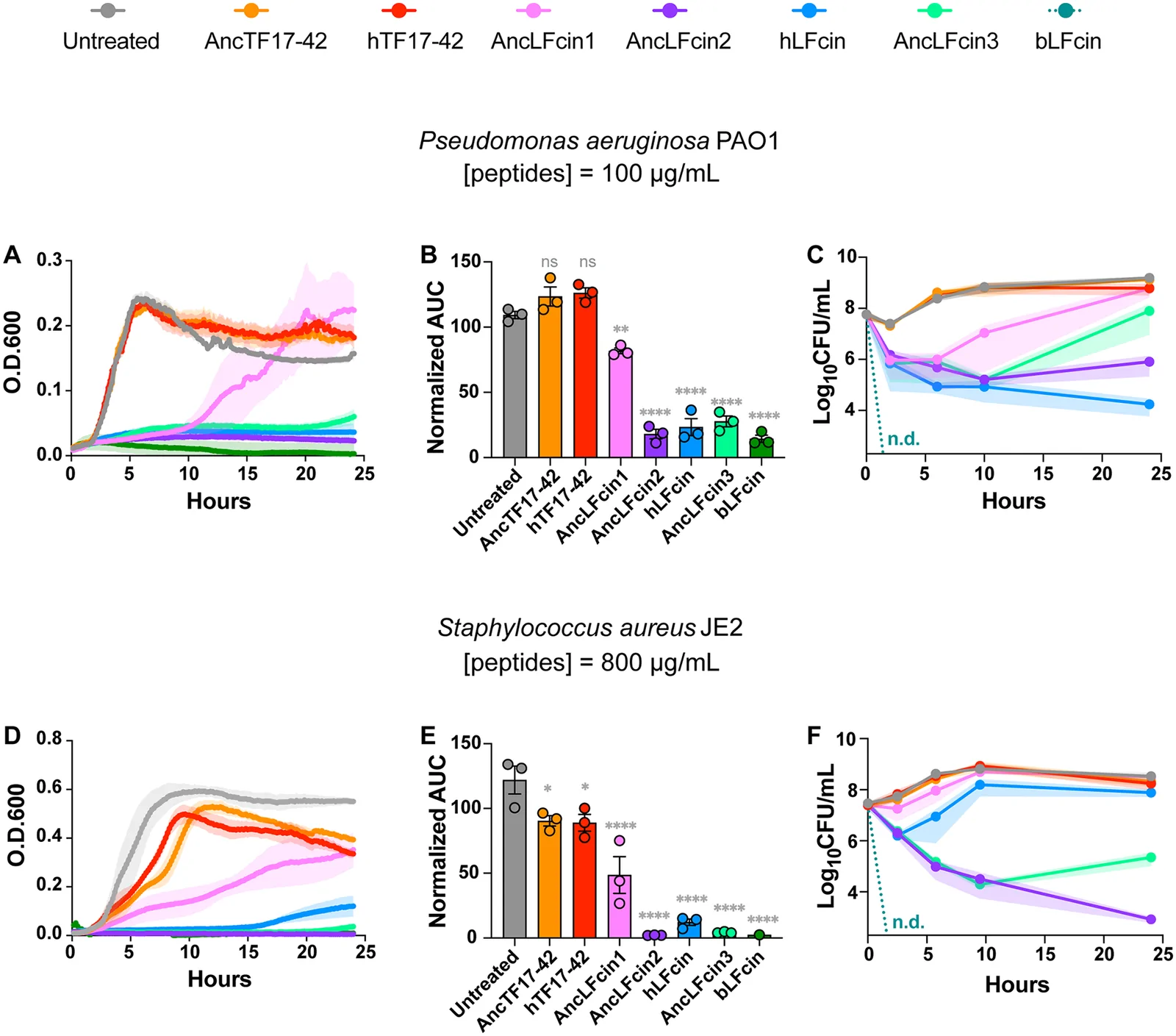
Ancestral lactoferricin possessed potent bactericidal activity. **(A–C)** *Pseudomonas aeruginosa* strain PAO1 treated with 100 μg/mL of indicated peptides, **(D–F)** *Staphylococcus aureus* strain JE2 treated with 800 μg/mL of indicated peptides. (A, D) Growth curves measured by optical density at 600 nm over time. (B, E) Corresponding area under the curve (AUC) measurements normalized to untreated control. (C, F) Bacterial survival measured as colony-forming units (CFUs) over the course of treatment. All data represent the mean ± SEM from three biological replicates. Statistical significance was determined using one-way ANOVA (*P ≤ 0.05, **P ≤ 0.01, ***P ≤ 0.001, ****P ≤ 0.0001; ns, not significant). The data underlying this Figure can be found in Tables H–K of S1 Data.

After broadly characterizing the antimicrobial nature of the ancestral and extant lactoferricins, we assessed their function in greater detail by measuring growth kinetics and viability of representative gram-negative (*P. aeruginosa*) and Gram-positive (*S. aureus*) bacteria in the presence of representative peptides (Fig 3). In addition to analyzing growth curves and calculating AUCs, we quantified bacterial survival by measuring colony-forming units (CFUs) over time. Based on the differences in dose-dependent activity observed previously, we used 100 µg/mL of the peptides against *P. aeruginosa* and 800 µg/mL against *S. aureus* in subsequent experiments.

Consistent with our previous results, extant and ancestral transferrin-derived peptides, hTF17–42 and AncTF17–42, respectively, exhibited no detectable effects on *P. aeruginosa* growth and survival (Fig 3A–3C). AncLFcin1 displayed an intermediate phenotype, with a modest but significant reduction in overall *P. aeruginosa* growth (Fig 3A and 3B). We also observed a rapid decrease in *P. aeruginosa* viability within 2 h of treatment, followed by a partial recovery (Fig 3C). In contrast, AncLFcin2 consistently suppressed *P. aeruginosa* growth throughout the experiment, resulting in a significant 1000-fold reduction in bacterial viability after 24 h of treatment (Fig 3A–3C). AncLFcin3, the ancestor of bLFcin, inhibited *P. aeruginosa* growth and caused 100–1,000 fold reduction in viability during the first 15 h of treatment, after which bacterial growth resumed. hLFcin also consistently suppressed *P. aeruginosa* growth, causing a 10,000 fold reduction in viability at the end of the treatment. Finally, bLFcin exhibited the most potent bactericidal effect, reflected by minimal AUC values and no detectable *P. aeruginosa* colonies within 2 h of treatment (Fig 3A–3C).

Lactoferricin activity profiles against *S. aureus* differed from those of *P. aeruginosa* in several ways (Fig 3D–3F). hTF17–42 and AncTF17–42 exhibited modest inhibitory effects on *S. aureus* later in the treatment period (Fig 3D and 3E), but these differences did not translate into reductions in bacterial viability as measured by CFU counts (Fig 3F). AncLFcin1 exerted partial inhibition against *S. aureus,* with little reduction in bacterial viability. AncLFcin2 strongly suppressed bacterial growth, resulting in an ~100,000 fold reduction in *S. aureus* viability by the end of treatment. AncLFcin3 also significantly inhibited *S. aureus* growth and reduced its viability over the course of the experiment. In contrast, hLFcin initially inhibited bacterial growth and caused a rapid 100 fold reduction in viability. Subsequently, *S. aureus* resumed growth, although this was not reflected in the optical density, possibly due to a reduction in cell size. Again, bLFcin remained the most potent peptide, eliminating detectable colonies within 2 h (Fig 3F). To assess these findings in alternative media environments, we also measured bacterial growth and survival in the presence of lactoferricin peptides in 0.5% milk and Opti-MEM media conditions (S5 Fig). While some variation relative to growth in TSB was observed, general trends of antimicrobial activity were consistent with previous results. Collectively, these findings indicate that weak antimicrobial activity emerged in the earliest lactoferricin ancestor, with bactericidal activity substantially enhanced in subsequent orthologs.

### Lactoferricin membrane permeabilizing activity arose early after gene duplication

**Fig 4:**
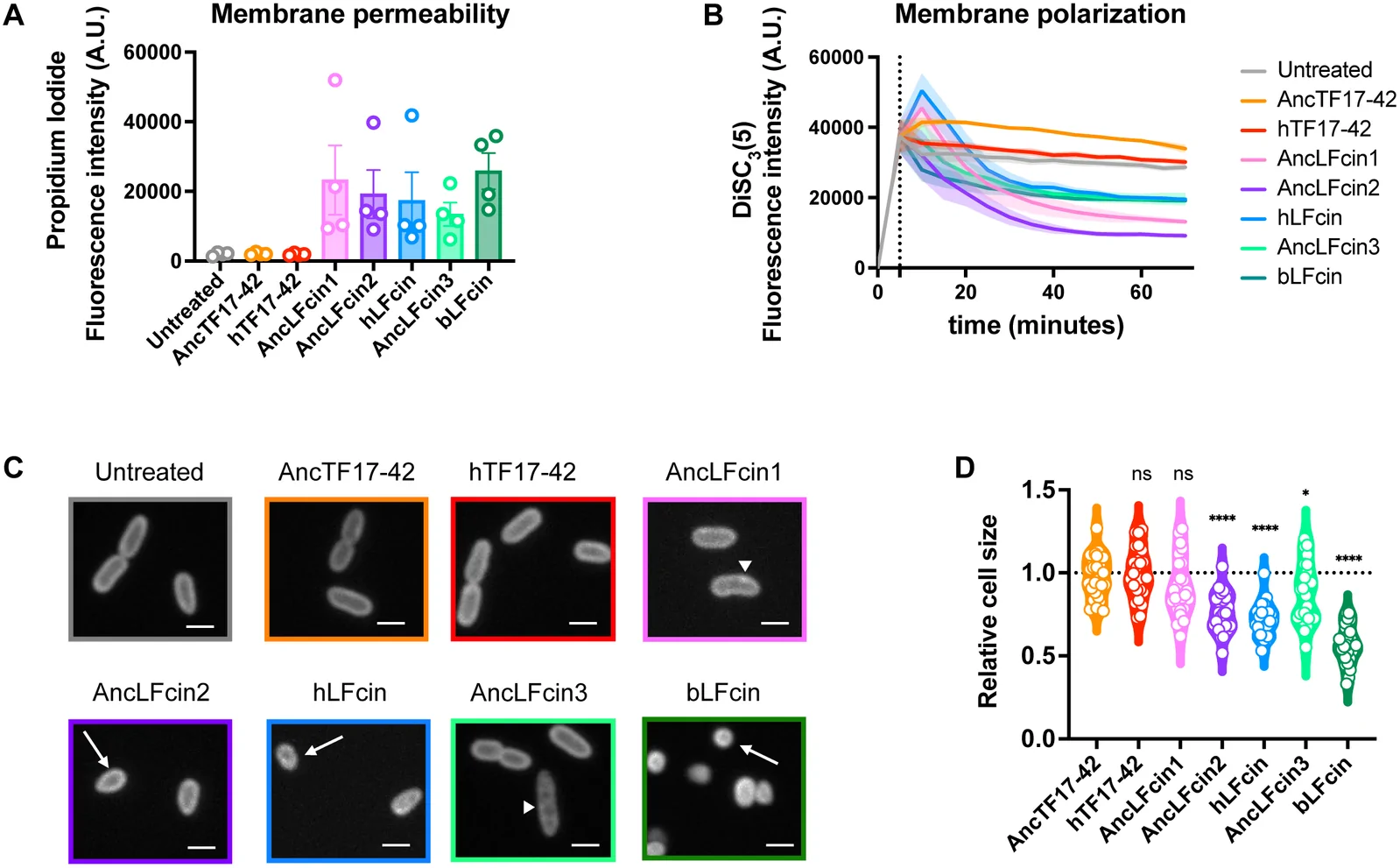
Lactoferricin membrane permeabilizing activity emerged early after gene duplication. *Pseudomonas aeruginosa* strain PAO1 was treated with 100 μg/mL of indicated peptides for 30–60 min to assess membrane disruption and cell morphology. **(A)** Membrane permeabilization measured by propidium iodide staining after 30 min of treatment. **(B)** Membrane polarization measured by DiSC3(5) dye after 1 h of treatment. Peptides were added 5 min after addition of the dye (shown with the dotted line). Lower fluorescence indicates hyperpolarization. Data represent mean and standard error based on three biological replicates. **(C)** Confocal microscopy images of peptide-treated cells at 30 min (scale bar, 1 μm). Triangles indicate Nile Red foci on bacterial membranes, suggesting increased membrane fluidity or puncta and arrows highlight abnormally shaped cells that are smaller and rounded. **(D)** Quantification of bacterial cell size from microscopy images (*n* = 100) on Fiji program. Statistical significance was determined using one-way ANOVA (*P ≤ 0.05, **P ≤ 0.01, ***P ≤ 0.001, ****P ≤ 0.0001; ns, not significant). The data underlying this Figure can be found in Tables J–N of S1 Data.

While both ancestral and extant lactoferricin peptides exhibited some degree of bactericidal activity, they varied dramatically in their potency (Fig 3). This led us to further examine the molecular mechanisms underlying differences in their activities. A common feature of many cationic AMPs is their ability to disrupt and permeabilize bacterial membranes [4,6]. To investigate this function, we measured the membrane permeabilizing ability of ancestral and extant lactoferricin peptides against *P*. *aeruginosa*. Membrane permeabilization was measured by staining with propidium iodide (PI) which enters the bacterial cell and binds DNA only when cell membranes are compromised [46]. We found that all ancestral and extant lactoferricins, but not transferrin-derived peptides, were capable of permeabilizing bacterial membranes within 30 min of incubation as detected by PI staining (Fig 4A). This finding indicates that bacterial membrane permeabilization was an early trait that emerged in the lactoferricin domain shortly after gene duplication.

Membrane permeabilization by AMPs can induce bacterial stress responses as well as alter membrane polarization and ion flux, which in turn impacts cell physiology and morphology [47,48]. To determine the extent to which ancestral and extant lactoferricins affect target cell physiology, we measured bacterial membrane polarization. *P. aeruginosa* cells were treated with the cationic dye 3,3′-dipropylthiadicarbocyanine iodide (DiSC_3_(5)) that accumulates in polarized membranes quenching its fluorescence [49]. Upon membrane depolarization, DiSC_3_(5) is released, and fluorescence increases. We found that all ancestral and extant lactoferricin-treated cells were hyperpolarized, as demonstrated by a reduction in fluorescence, compared to untreated, hTF17–42, or AncTF17–42-treated cells (Fig 4B).

Given that all lactoferricin peptides exhibited both membrane permeabilizing and polarization-altering activities, we sought to understand how these peptides nonetheless differed in their overall antimicrobial potency. To address this, we shifted from population-level assays to single-cell morphological characterization. We hypothesized that lactoferricin variants with increased antimicrobial activity would exert greater impact on bacterial cell envelope integrity. To visualize this, we treated *P. aeruginosa* with the lipophilic dye Nile Red, which homogenously binds to membranes and produces brightly stained foci in regions of increased fluidity, indicative of membrane perturbation [46]. Using spinning-disc confocal microscopy, hTF17–42 and AncTF17–42 exhibited no visible effects on bacterial morphology (Fig 4C). In contrast, cells treated with AncLFcin1, AncLFcin2, or AncLFcin3 displayed puncta-like structures on their membranes, suggesting localized membrane disturbance. These cells also appeared significantly smaller than untreated controls. This effect was most pronounced with hLFcin and bLFcin, where cells became markedly reduced in size, rounded, and membranes were barely distinguishable from the cytosol (Fig 4C). Quantitative measurements of cell size confirmed that ancestral lactoferricins induced moderate but significant cell shrinkage, whereas extant variants amplified this effect (Fig 4D).

Membrane disruption is a common mechanism of AMP function, which can cause leaking of cytoplasmic content and shrinkage of cells [46,50,51]. Such perturbations can be transient and resealable or irreversible, depending on the extent of the damage [51,52]. Our results suggest that bacteria are able to recover from ancestral lactoferricin-induced membrane damage, whereas extant variants, particularly bLFcin, caused irreversible cell envelope collapse. Together, these findings indicate that the lactoferricin domain initially possessed membrane permeabilizing activity, which intensified during evolution to produce potent bactericidal effects.

### A single arginine substitution contributed to the early emergence of lactoferricin antimicrobial activity

**Fig 5:**
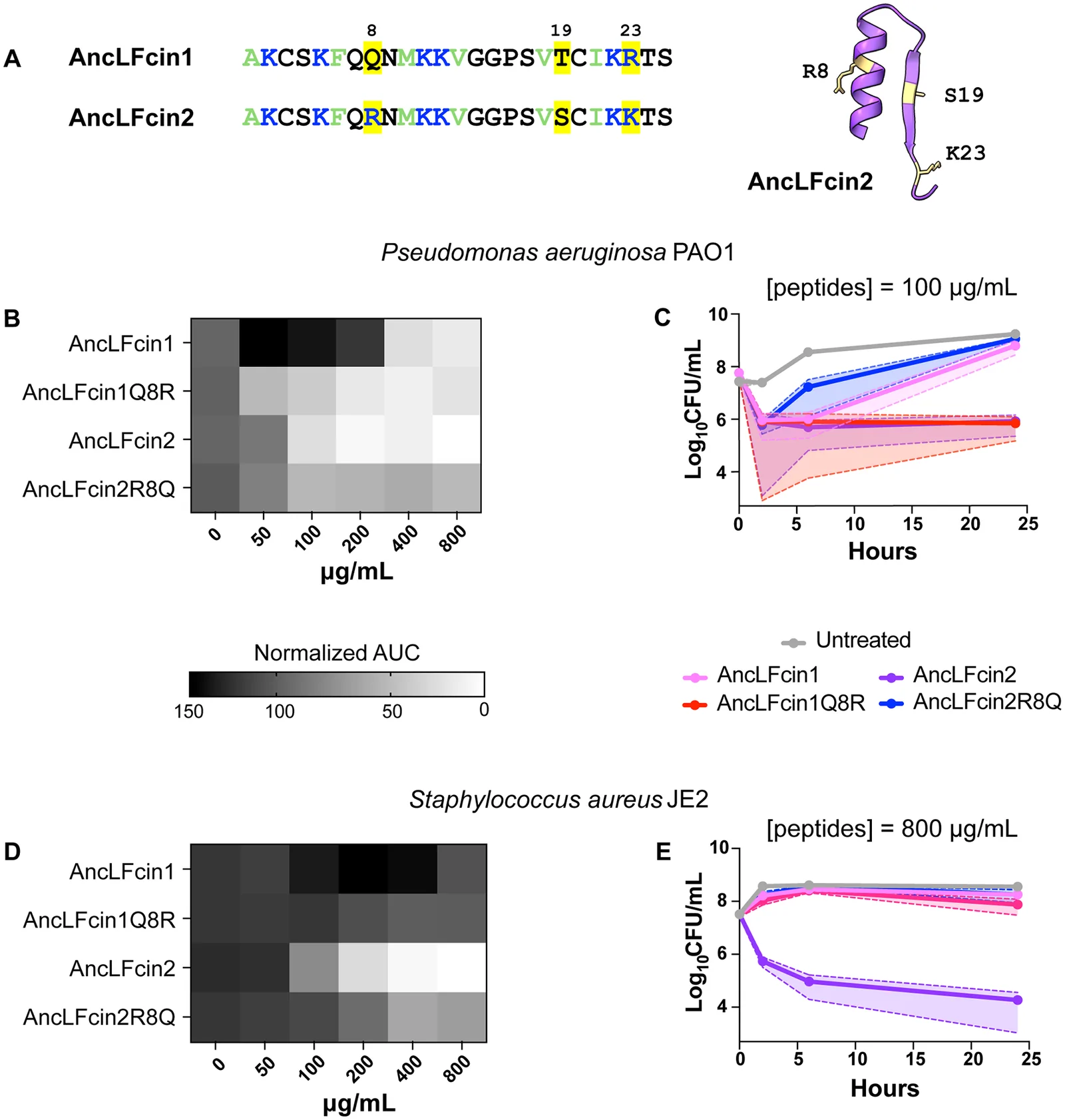
A single arginine substitution contributed to the early emergence of lactoferricin antimicrobial activity. **(A)** Sequences of AncLFcin1 and AncLFcin2. Amino acids are colored by character: hydrophobic (green), cationic (blue), and neutral (black). Residues that differ between AncLFcin1 and AncLFcin2 are highlighted in yellow, both in the sequences and on the predicted AncLFcin2 structure. Variants were generated in each background: AncLFcin1Q8R and AncLFcin2R8Q. The antimicrobial activity of AncLFcin1, AncLFcin1Q8R, AncLFcin2, and AncLFcin2R8Q was compared against *Pseudomonas aeruginosa* PAO1 **(B, C)** and *Staphylococcus aureus* JE2 **(D, E)**. **(B, D)** Area under the growth curve (AUC) at the indicated peptide concentrations, normalized to the mean AUC of untreated cultures (no peptide). **(C, E)** Bacterial survival, measured as colony-forming units (CFU), at the indicated concentration over the course of treatment. Data represent mean and standard error from three biological replicates. The data underlying this Figure can be found in Tables O–S of S1 Data.

We noted that the ancestral lactoferricin sequences AncLFcin1 and AncLFcin2 vary substantially in their activity, but only differ at three amino acid positions (Figs 3 and 5A). One of these, position 8, introduces a cationic arginine residue in AncLFcin2. To determine whether this single substitution contributed to the early evolution of lactoferricin antimicrobial activity, we generated two additional mutant peptides in which arginine (R) 8 is substituted for glutamine (Q) in AncLFcin1 and the reciprocal arginine to glutamine substitution is made in AncLFcin2. Measuring bacterial growth as well as CFUs over time, we observed that the arginine substitution at position 8 was both necessary and sufficient to confer enhanced antimicrobial activity against *P. aeruginosa* (Fig 5B and 5C). In contrast, while arginine at position 8 was also necessary for AncLFcin2 to inhibit *S. aureus* strain JE2, this substitution was not sufficient to increase the activity of AncLFcin1 (Fig 5D and 5E). These findings indicate that the early Q8R substitution played an important role in the increased activity of ancestral lactoferricin. In addition, the differences observed between *P. aeruginosa* and *S. aureus* demonstrate how AMP genetic variation can have variable effects against different microbes.

### Natural selection modulated lactoferricin antimicrobial activity in primates

**Fig 6:**
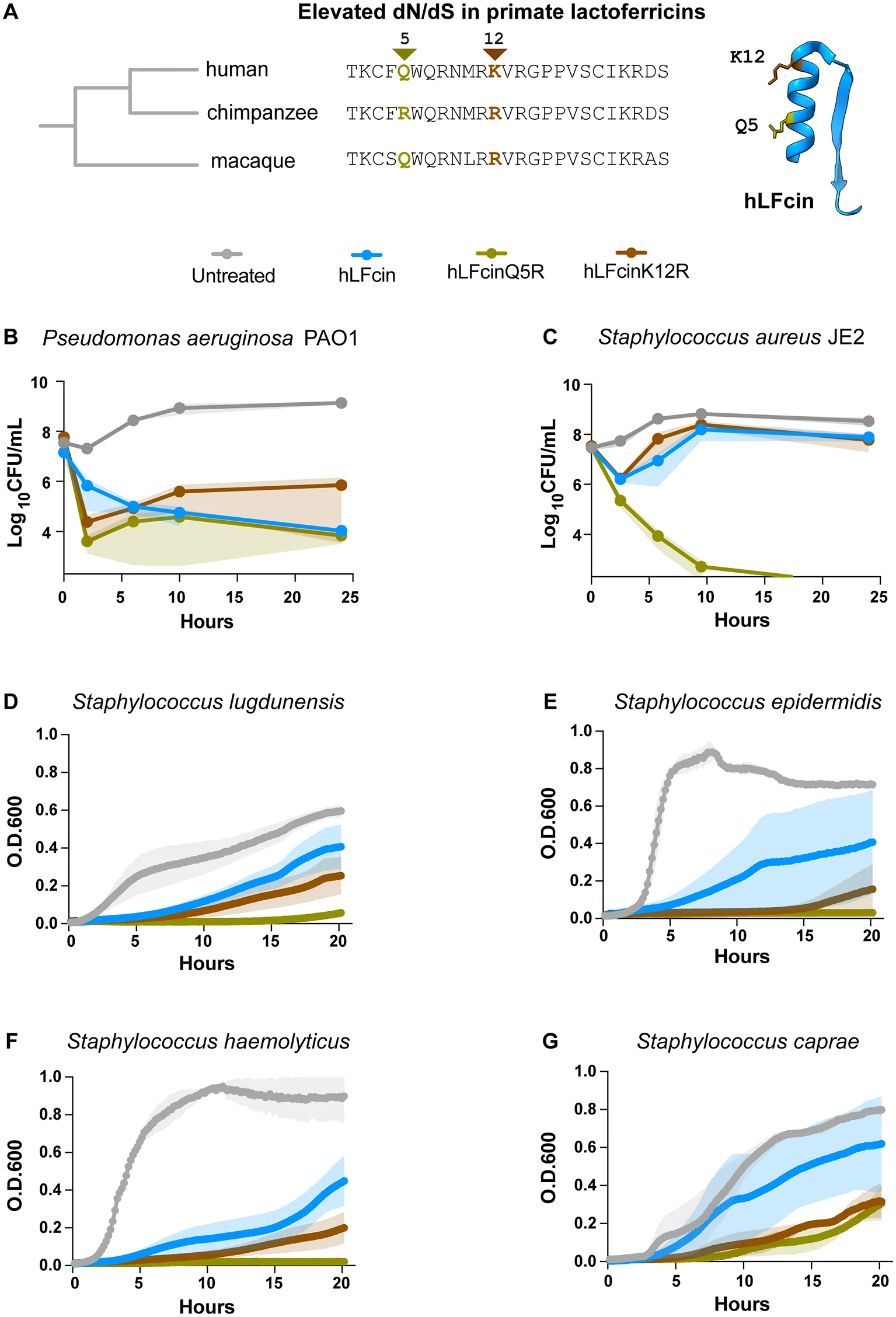
Natural selection modulates lactoferricin antimicrobial potency in primates. **(A)** Alignment of lactoferricin orthologs from human, chimpanzee, and rhesus macaque. Two sites (positions 5 and 12) showed evidence of diversifying selection (dN/dS > 1) across primates as reported previously. Two hLFcin variants were synthesized, each carrying a single substitution at one of these sites: hLFcinQ5R and hLFcinK12R. The antimicrobial activity of hLFcin and the two variants was measured as colony-forming units (CFU) against *Pseudomonas aeruginosa* PAO1 at 100 μg/mL **(B)** and *Staphylococcus aureus* JE2 at 800 μg/mL **(C)**. Activity was further screened by monitoring bacterial growth (optical density) against additional *Staphylococcus* species: *S. lugdunensis* **(D)**, *S. epidermidis* **(E)**, *S. haemolyticus* **(F)**, and *S. caprae* **(G)**. Data represent the mean ± SEM from three biological replicates. The data underlying this Figure can be found in Tables T–Y of S1 Data.

In addition to investigating the early origins of the lactoferricin domain, we also sought to determine how lactoferricin antimicrobial activity has changed over recent evolutionary timescales. Previous work by our group found evidence that lactoferrin has been subject to repeated positive selection within human populations as well as between simian primate species, suggesting antimicrobial activity may have been modulated via recurrent adaptation [18,33]. We identified several sites in the N-lobe of lactoferrin with elevated rates of nonsynonymous to synonymous substitutions (dN/dS), including two within the lactoferricin region (Figs 6A and S6A) [33]. For example, position 5 varies between primates, with humans and many monkeys encoding a glutamine, whereas all other great apes encode an arginine. In addition, position 12 is variable among primates as well as polymorphic within human populations, generally toggling between cationic arginine and lysine (R12 or K12) residues [33].

To assess the functional consequences of natural selection at sites 5 and 12, we generated two point mutants in human lactoferricin: hLFcinQ5R and hLFcinK12R. hLFcinK12R exhibited antimicrobial activity comparable to wild type hLFcin against both *P. aeruginosa* PAO1 and *S. aureus* JE2*,* indicating that the K12R substitution does not substantially alter survival of these pathogens (Fig 6B and 6C). In contrast, the hLFcinQ5R mutant displayed significant enhancement in antimicrobial potency against *S. aureus* relative to hLFcin (Fig 6B and 6C). To further investigate these differences, we treated four distinct *Staphylococcus* isolates with each of these four peptides and measured growth over time. Consistent with our earlier experiments, the hLFcinQ5R peptide suppressed growth of *S. lugdunensis, S. epidermidis, S. caprae, and S. capitis* (Figs 6D–6G and S6B–S6E). In addition, we noted a modest but consistent growth reduction for bacteria treated with hLFcinK12R relative to hLFcin, suggesting that genetic variation at this position may have subtle effects on lactoferricin antimicrobial activity. This trend was also consistent when performed in Opti-MEM media (S7 Fig). Taken together, these findings indicate that antimicrobial activity of the lactoferricin domain has been repeatedly modulated by natural selection in primates.

## Discussion

AMPs have long been appreciated as deeply conserved effectors of innate immunity. Recent large-scale analyses further suggest that AMP-like domains are far more widespread in plant and animal proteomes than previously appreciated [10–13]. The existence of such cryptic antimicrobial functions suggests a previously unappreciated layer of host defense, though the evolutionary origins of embedded AMPs remain largely uncharacterized. Leveraging mammalian lactoferrin and its embedded AMP lactoferricin as a model, we observed that even the earliest lactoferricin ancestor possessed the ability to permeabilize bacterial membranes and alter membrane potential, a property that was further enhanced in later ancestors (Fig 4). This increased activity was accompanied by a marked perturbation of bacterial cell morphology, likely due to leakage of cytoplasmic content [50–52]. Membrane permeabilization by AMPs is often reversible depending on the severity of damage [51,52]. Hence, it is reasonable to speculate that early lactoferricin domains induced perturbations that bacteria could repair, allowing eventual recovery. In contrast, more potent lactoferricin variants such as bLFcin induced irreversible damage at much lower concentrations, leading to complete lysis or severe morphological damage that prevented recovery (Fig 4). These findings demonstrate that membrane permeabilization and hyperpolarization were early antimicrobial traits of lactoferricin, and that bactericidal activity intensified over time. Assessing the activity of additional lactoferricin ancestors or mutants could aid in further resolving the molecular basis of this activity in future work.

We noted that some ancestral lactoferricins exerted higher antimicrobial activity than their extant orthologs. For example, AncLFcin2 and AncLFcin3 were consistently more bactericidal against *S. aureus* than human lactoferricin (Figs 2 and 3). Thus, the evolution of antimicrobial function in lactoferrin does not reflect a simple linear increase in potency but has rather fluctuated along different lineages depending on the target microbe. We considered whether a reduction in lactoferricin antimicrobial activity could indicate a trade-off to minimize toxicity against host cells. However, treatment of bovine erythrocytes revealed no significant hemolysis in the presence of any lactoferricin peptides, inconsistent with this hypothesis (S8 Fig). An alternative explanation is that some bacteria may have evolved resistance to particular host-derived AMPs. In addition, trade-offs with antimicrobial activity could arise due to other factors such as peptide stability, ease of protease digestion, interactions with bacterial lactoferrin receptors, or iron-binding ability of the N-lobe. We note that the majority of these experiments were performed in a low-nutrient laboratory media which do not fully recapitulate the host environment, although general trends in activity were conserved in other media environments (S5 Fig). Past studies have demonstrated how factors such as pH or ionic environment can impact the effectiveness of AMPs in vitro and in vivo [53,54]. Such environmental factors would also be expected to influence the evolution of AMPs. Future studies could aid in understanding how host chemical environments shape the emergence of AMP functions. Together, these results underscore how ASR can uncover unique variants of extant AMPs that could serve as safe and effective pathogen treatment options, particularly in the era of growing antibiotic resistance [5,55].

AMPs have frequently been identified among the most rapidly evolving genes in animal genomes, likely reflecting repeated adaptation in response to diverse microbes [56,57]. In contrast, far less is known about how AMP domains embedded within larger proteins emerge and evolve in response to selection. We previously identified two sites exhibiting signatures of positive selection in the lactoferricin domain among primates [33]. Notably, one of these substitutions (Q5R) was sufficient to significantly enhance the antimicrobial activity of human lactoferricin, particularly against *S. aureus* (Figs 6, S6 and S7). This observation suggests the importance of this region for lactoferricin’s activity. Notably, the adjacent sixth residue is invariably an aromatic hydrophobic amino acid, either phenylalanine (F) or tryptophan (W), across all lactoferricin sequences (Fig 1C). The most potent variant tested, bovine lactoferricin, contains three such pairs of cationic and aromatic residues (F1K2, R5W6, and W7R8). Given that cationic nature and hydrophobicity are key features of many AMPs and other antimicrobial proteins [4,58], the juxtaposition of a cationic residue with an aromatic hydrophobic residue within lactoferrin could reflect epistasis between these sites. Indeed, epistasis between nearby R and W residues has been reported previously in antiviral proteins [59]. It is well established that cationic amino acids mediate electrostatic interactions with the negatively charged microbial surfaces, whereas hydrophobic residues insert into membranes and contribute to disruption of the lipid bilayer [60]. Future work could resolve how selection and epistasis together contribute to antimicrobial potency in these and other AMPs.

It is notable that lactoferrin has been reported to contain not just one, but multiple embedded antimicrobial domains. For example, the lactoferrin N-lobe contains a 17-amino acid region termed lactoferrampin that possesses antimicrobial activity against both bacteria and fungi [61]. In addition, ovotransferrin, the ortholog of mammalian transferrin in birds, has also been reported to contain an antimicrobial fragment within the N-lobe in a region distinct from both lactoferricin and lactoferrampin [62,63]. These observations suggest that embedded AMPs may have arisen multiple times within lactoferrin or transferrin homologs during animal evolution. This protein family could thus provide an informative system to further investigate the origins and evolution of antimicrobial functions.

In addition to providing protection against invasive pathogens, the expression of AMPs at barrier tissues positions them to broadly shape the host-associated microbiota. Work in *Drosophila* has demonstrated how natural selection and polymorphisms in AMP genes can impact diverse resident microbes [56,64,65], as well as confer defense against microbes within specific ecological niches [66]. Given that lactoferrin is among the most abundant secreted proteins in many mammalian barrier tissues, lactoferrin genetic variation may similarly shape the diversity and composition of host-associated microbial communities. By retracing the origin and evolution of antimicrobial activity in mammalian lactoferrin, our study illustrates how new protein functions can emerge to promote host defense against major microbial pathogens.

## Materials and methods

### ASR

Ancestral sequences were reconstructed using the Topiary pipeline [37]. To perform the reconstruction including early vertebrate transferrins, cartilaginous fish were used as the outgroup. The input sequences collected from UniProt were transferrins from *Perca flavescens* (A0A484DEK0), *Danio rerio* (Q6P3G0), *Gallus gallus* (P02789), and *Homo sapiens* (P02787); lactoferrins from *Desmodus rotundus* (K9IMD0), *Bos taurus* (P24627), and *Homo sapiens* (P02788); and melanotransferrins from *Ornithorhynchus anatinus* (F6Z643) and *Homo sapiens* (P08582). Briefly, Topiary used the seed sequences to perform a BLAST search against the NCBI nonredundant protein database within the defined taxonomic scope and generated a multiple sequence alignment (MSA). The alignment was manually inspected to remove the indels and gap regions. Topiary then inferred a maximum-likelihood gene tree, reconciled it with the species tree, and reconstructed ancestral sequences at every node. For each node, Topiary generates the maximum-likelihood sequence alongside an “Altall” variant, in which an alternative residue was substituted at ambiguously reconstructed positions. Ambiguity was assigned using Topiary’s default posterior probability cutoff of 0.25.

### Peptide synthesis

All peptides were commercially synthesized by GenScript. Lyophilized peptides were resuspended in phosphate-buffered saline (PBS, pH 7.0) to 10 mg/mL and stored at –80 °C. These stock solutions were diluted to desired concentrations in water as needed, while minimizing freeze-thaw cycles.

### Structural prediction of ancestral sequences

Protein structures for the reconstructed ancestral sequences were predicted using ColabFold, a streamlined pipeline of AlphaFold2 [67]. Prior to prediction, the N-terminal region corresponding to the human lactoferrin signal peptide (hLF1–19) was removed from all ancestral sequences. Predicted protein structures were visualized and analyzed using UCSF ChimeraX (version 1.8) [68]. The theoretical isoelectric point (pI) of each peptide was calculated using the ExPASy Compute pI/Mw tool [69].

### Bacterial growth assays

Overnight cultures of all bacterial strains were prepared by inoculating a single colony into low-nutrient 5% tryptic soy broth (TSB, v/v) to model the low-nutrient conditions of the host environment and incubated at 250 rpm overnight, unless otherwise specified. The following day, cultures were diluted to an optical density at 600 nm (OD₆₀₀) of 0.02 in fresh 5% TSB or in Opti-MEM where specified. For growth assays, 150 μL of the diluted culture was dispensed into each well of a 96-well microtiter plate, followed by the addition of peptides at the indicated concentrations. Plates were incubated at 37 °C for 24 h with continuous shaking in a Synergy H1 microplate reader (BioTek), and bacterial growth was monitored by measuring OD₆₀₀ at 10-min intervals. OD₆₀₀ values were blank-corrected by subtracting the OD₆₀₀ of cell-free wells containing an equivalent volume of medium with the matching peptide concentration (or no peptide) from that of the corresponding culture wells. The area under the curve (AUC) was calculated and normalized on GraphPad Prism [Version 11.0.2]. For normalization of the AUCs in the GraphPad software, 100% was defined as the mean AUC of untreated bacterial cultures (no peptide) and 0% as an AUC of zero (no bacterial growth).

### Bacterial survival assay

Overnight cultures were diluted to an OD₆₀₀ of 0.02 in fresh medium (5% TSB or 0.5% milk (w/v) as specified), and antimicrobial peptides were added at the indicated concentrations in 1.5 mL microcentrifuge tubes. To assess bacterial viability over time, samples were taken at defined time points during the treatment period. At each time point, cultures were serially diluted 10-fold (up to 10^−7^) and plated on tryptic soy agar (TSA) to enumerate CFUs. Plates were incubated at 37 °C overnight, and CFUs were counted the following day to evaluate the time-course of bacterial survival.

### Membrane permeabilization measurements

Overnight cultures of *Pseudomonas aeruginosa* PAO1 were subcultured by diluting to an OD₆₀₀ of 0.3 in fresh 5% TSB. Aliquots of 100 μL were transferred into 1.5 mL microcentrifuge tubes and treated with the peptides for 30 min. Cells were pelleted by centrifugation at 5,000  *g* for 1 min. Pellets were resuspended in 300 μL of 20 μM PI (Sigma-Aldrich P4170-10MG) and incubated in the dark for 15 min to prevent photobleaching. Excess dye was removed by centrifugation at 5,000  *g* for 1 min, the supernatant was discarded, and pellets were resuspended in 300 μL of PBS (pH 7.0). For fluorescence measurement, 100 μL aliquots from each sample were transferred in triplicate into black-walled, clear-bottom 96-well plates (without lids). Fluorescence intensity was measured from the bottom of the plate using a microplate reader with excitation at 535 nm and emission at 617 nm.

### Membrane potential measurements

The membrane depolarizing activity of peptides was assessed using the voltage-sensitive dye DiSC_3_(5) (MedChem Express #HY-D0085-25 mg) as previously described [49]. *Pseudomonas aeruginosa* PAO1 overnight cultures were diluted in fresh 5% TSB to OD₆₀₀ of 0.3. DiSC_3_(5) [49], prepared as a stock solution in DMSO, was added to the bacterial suspension at a final concentration of 5 μM. Following a brief equilibration period to allow dye accumulation across polarized membranes, peptides were added at a final concentration of 100 μg/mL in a black, clear-bottom 96-well plate. Fluorescence was monitored kinetically over a 1-h period at 37 °C using a Synergy H1 microplate reader (BioTek), with excitation and emission wavelengths set to 610 nm and 660 nm, respectively. A decrease in fluorescence intensity was interpreted as membrane hyperpolarization, reflecting increased accumulation of the cationic, lipophilic dye within the bacterial cytoplasmic membrane, where self-quenching reduces the overall fluorescence signal.

### Nile Red staining, microscopy and cell size measurements

The Nile Red staining protocol was adapted and modified from Dombach *and colleagues*, 2021 [70]. Overnight bacterial cultures were diluted in fresh 5% TSB (v/v) to an optical density at 600 nm (OD₆₀₀) of 0.3. From this, 100 μL aliquots were transferred to 1.5 mL microcentrifuge tubes for peptide treatment. Samples were incubated at 37 °C for 40 min with shaking at 250 rpm. Following peptide treatment, Nile Red (Sigma-Aldrich 72485-100MG) was added to each tube at a final concentration of 10 μg/mL, followed by a 5-min incubation at 37 °C. Cells were then fixed by adding paraformaldehyde to a final concentration of 4%, and incubated at room temperature for 10 min. After fixation, cells were pelleted by centrifugation at 10,000  *g* for 1 min and resuspended in 10 μL of PBS, pH 7.0. For microscopy, 7 μL of the resuspended cells was applied to a coverslip, overlaid with 0.5% agarose, and gently compressed with a glass slide to immobilize the sample. Imaging was performed using a SoRA spinning disk confocal microscope (Nikon CSU-W1) equipped with a 60X water-immersion objective. Bacterial cell size was measured using the Fiji package of ImageJ software [71].

### Hemolysis assay

The hemolysis assay was adapted from Greco and colleagues, 2020 [72]. Bovine red blood cells (RBCs) were isolated by centrifuging 1 mL of whole blood at 3,000  *g* for 2 min. The RBC pellet was washed five times with PBS by resuspension and centrifugation until the supernatant was clear, then diluted 1:10 in PBS. In a 96-well plate, 100 µL of the diluted RBC suspension was mixed with each peptide solution (final concentration: 1 mg/mL) and incubated at 37 °C for 1 h. Absorbance was measured at 405 nm using a microplate reader, and the percentage of hemolysis was calculated relative to a positive control (1% Triton X-100).

## Supporting information

S1 Fig Functional divergence of mammalian lactoferrin and transferrin. **(A)** The extant lactoferrin (PDB: 1lfg) and serum transferrin (PDB: 3qyt) genes arose via duplication of transferrin in the ancestor of placental mammals. Surface electrostatic potential is shown. While transferrin maintains key roles in iron-binding and transport, lactoferrin has acquired new functions including direct bactericidal activity. **(B)** Antimicrobial activity of extant human and bovine lactoferricin (hLFcin and bLFcin, respectively) against *Pseudomonas aeruginosa* PAO1 compared with the equivalent region in human transferrin (hTF17-42). The data underlying this Figure can be found in Table Z of S1 Data.
(TIF)

S2 Fig Ancestral sequence reconstruction (ASR) of vertebrate transferrin family proteins. (A) Amino acid alignment of the 25-amino acid stretch of lactoferricin region across placental mammals. Blue, red, and green letters denote positively charged, negatively charged, and hydrophobic aromatic residues, respectively. Species tree was adapted from Murphy and colleagues 2001 [73]. (B) A simplified tree showing the scope of ASR used in this study. This tree shows the representative taxa from which transferrin homologs were used to reconstruct the ancestral sequences. A total of 376 sequences of transferrin, lactoferrin, and melanotransferrin from diverse vertebrate species were used as input in this process. AncTF highlights the last ancestral transferrin from the reconstruction, after which lactoferrin arose via gene duplication in placental mammals. The data underlying this Figure can be found in S2 Data.
(TIF)

S3 Fig Reconstructed ancestral sequences and alternate variants. Posterior probabilities of each site are shown for (A) AncTF, (B) AncLF1, (C) AncLF2 and (D) AncLF3. Blue indicates the most probable amino acid at each position, while red represents the *alt_all* sequence containing an alternate amino acid at ambiguously reconstructed sites (posterior probability cutoff for ambiguity is 0.25). The lactoferricin regions are highlighted with cyan rectangles in the full-length sequences. (E) Sequences of the lactoferricin regions in the reconstructed ancestors. Yellow highlight denotes residues at the ambiguous positions that differ between the most probable (blue) and *alt_all* (red) sequences. The data underlying this Figure can be found in Tables AA–AD of S1 Data.
(TIF)

S4 Fig Antimicrobial activities of alternate ancestral peptides. Heatmaps show area under the curve (AUC) values normalized against the untreated control from bacterial growth curves in the presence of the indicated peptides at specified concentrations. *Pseudomonas aeruginosa* PAO1 is represented in pink, *Staphylococcus aureus* JE2 is shown in purple. The data underlying this Figure can be found in Tables AE and AF of S1 Data.
(TIF)

S5 Fig Lactoferricin peptide activity in alternative media conditions. **(A, B)** Bacterial survival measured as colony-forming units (CFUs) over the course of treatment in 0.5% milk solution. *Pseudomonas aeruginosa* strain PAO1 was treated with 100 μg/mL of indicated peptides, *Staphylococcus aureus* strain JE2 treated with 800 μg/mL of indicated peptides. (**C, E**) Growth curves measured by optical density at 600 nm over time in Opti-MEM media. (**D, F**) Corresponding area under the curve (AUC) measurements normalized to untreated control. All data represent the mean ± SEM from three biological replicates. Statistical significance was determined using one-way ANOVA (*P ≤ 0.05, **P ≤ 0.01, ***P ≤ 0.001, ****P ≤ 0.0001; ns, not significant). The data underlying this Figure can be found in Tables AG–AK of S1 Data.
(TIF)

S6 Fig Evolution of lactoferricin across simian primates. **(A)** Lactoferricin positions 5 and 12 exhibited elevated dN/dS consistent with repeated positive selection. In great apes, glutamine (Q) at position 5 is replaced by arginine (R), adjacent to a tryptophan (W). At position 12, residues vary between arginine (R) and lysine (K) across primate species. CEmacaque: crab-eating macaque; Tmacaque: long-tailed macaque; SN_monkey: snub-nosed monkey; squirrelM: squirrel monkey; woolly: wolly monkey. Species tree was adapted from Barber and colleagues 2016 [33]. **(B–E)** Heatmaps show area under the growth curve (AUC) values, normalized to the untreated control, for the indicated peptides at the indicated concentrations against different *Staphylococcus* species. Each cell represents the mean of three biological replicates. The data underlying this Figure can be found in Tables V–Y of S1 Data.
(TIF)

S7 Fig Bacterial growth measurements in the presence of lactoferricin mutants. Growth curves measured by optical density at 600 nm over time in Opti-MEM media. Data represent the mean ± SEM from three biological replicates. The data underlying this Figure can be found in Table AL of S1 Data.
(TIF)

S8 Fig Hemolysis of ancestral and extant peptides. Bovine red blood cells (RBCs) were treated with peptides at 1 mg/mL. Complete lysis (100% hemolysis) was achieved with 1% Triton X-100 as a positive control. Hemolysis by the peptides is shown as a percentage relative to this control. Data represent three biological replicates with the standard error. The data underlying this Figure can be found in Table AM of S1 Data.
(TIF)

S1 Data Raw data for Figs 1–6 and S1–S8. (XLSX)

S2 Data Newick file of the ancestral sequence reconstruction tree. The tree contains 376 vertebrate sequences of transferrin (TF), lactoferrin (LTF), and melanotransferrin (MLTF) used to infer the ancestral sequences.
(TXT)

S3 Data Reconstructed ancestral sequences used for analysis. This file contains the full-length amino acid sequences of AncTF, AncLF1, AncLF2, and AncLF3, including both their most likely sequences and the *alt_all* variants, in which each position is assigned the second most likely amino acid. The average posterior probability (PP), number of ambiguous sites (num_ambig), and number of ambiguous gaps (num_ambig_gaps) for each sequence are provided in the sequence headers. The magenta-highlighted region (first 19 amino acids) represents the predicted signal peptide, which was removed prior to structural visualization. The cyan-highlighted region corresponds to the human lactoferricin-equivalent segment of 25 amino acids, which was used for experimental purposes to test the evolutionary development of antimicrobial activity within the lactoferricin domain.
(DOCX)

## Abbreviations

ASR: Ancestral sequence reconstruction
AUC: area under the curve
CFUs: colony-forming units
MSA: multiple sequence alignment
PBS: phosphate-buffered saline
PI: propidium iodide
PP: posterior probability
RBCs: red blood cells
TSA: tryptic soy agar
TSB: tryptic soy broth.
