# Supplementary material for: Retracing the origin and evolution of a cryptic antimicrobial peptide within mammalian lactoferrin
Source: PLoS Biol. 2026 Aug 25;24(8):e3003932. doi: 10.1371/journal.pbio.3003932 (PMC13505947; doi:10.1371/journal.pbio.3003932)
Supplement: Supplementary file 2 [file pbio.3003932.s015.pdf]

June 23, 2026

Dear editors,

Thank you for the opportunity to provide a revised manuscript for consideration at *PLOS Biology*. We have updated the manuscript with a number of new experimental results (particularly in revised Figures 5 and 6), as well as extensive text editing to address all editor concerns. Please find our responses to the academic editor comments below in **bold.**

COMMENTS FROM THE ACADEMIC EDITOR:

“Stepwise origin and evolution of a cryptic antimicrobial peptide in mammalian lactoferrin” by Sil et al.

The manuscript presents an integrative ancestral reconstruction and functional analysis of the lactoferricin domain of mammalian lactoferrin, documenting how cationic and hydrophobic features and antimicrobial potency have changed over deep time and on recent primate timescales. The work is clearly written, technically competent, and addresses questions of broad interest in protein evolution, innate immunity, and host–pathogen interactions. However, given the detailed prior peer review and the current framing, some revisions are required before further consideration.

Major points to address- Experimental

1. (Required) Test charge/hydrophobicity hypothesis with minimal mutant panel (AncLFcin1↔AncLFcin2) Directly connect the key substitutions between AncLFcin1 and AncLFcin2 to the observed jump in antimicrobial potency, strengthening the “stepwise tuning” narrative reviewers already engaged with. For this, we propose that you synthesize and test AncLFcin1_Q8R, AncLFcin1_T19S, and AncLFcin1_R23K (as you already proposed to PNAS). Alternatively, or in addition, you can synthesize and test the reciprocal mutations in AncLFcin2 (R8Q, S19T, K23R) to see if you can partially “revert” activity. You would be expected to carry out the same growth/AUC + CFU assays as Fig. 2–3 against one Gram negative (e.g., PAO1) and one Gram positive (e.g., S. aureus MN8). You could also assay the membrane permeabilization (PI uptake) at a single dose to see whether the major effect is on binding/permeabilization vs downstream killing. This will directly test your central mechanistic hypothesis (incremental gains in positive charge/hydrophobic packing drive increased antimicrobial activity), rather than inferring it from correlational ancestral reconstructions. We believe that this experiment provides a clean, minimalist “evolutionary swap” experiment that is

reviewer friendly and squarely within your research protocols (short synthetic peptides in standard assays

We opted to test the activity of AncLFcin1_Q8R and the AncLFcin2_R8Q peptides given our hypothesis that introduction of positively charged amino acids has played an important role in the evolution of lactoferricin antimicrobial activity. We performed both growth curve/AUC experiments as well as generated survival curves by measuring CFUs over time. These data are reported in revised Figure 5. We find that the single substitution at position 8 is both sufficient and necessary to enhance antimicrobial activity against *P. aeruginosa*. Notably, while the single R to Q substitution is necessary to reduce antimicrobial activity of AncLFcin2, this substitution is not sufficient to increase activity of AncLFcin1 against *S. aureus* strain JE2. These results suggest that the single Q to R substitution at position 8 played an important role in the early evolution of lactoferricin antimicrobial activity. Notably, the reduced antimicrobial activity of AncLFcin1_Q8R against *S. aureus* demonstrates that antimicrobial peptide mutations exhibit variable effects against different microbes. This is also consistent with findings from our previous mutant peptides now reported in revised Figure 6. We have revised the Results and Discussion to acknowledge these points. Figure 6 also includes new data testing the effects of mutant peptides against a wider range of *Staphylococcus* strains, demonstrating the effects of rapidly-evolving sites across a wider range of bacteria. Collectively we believe these additional experiments provide a much improved understanding of lactoferricin evolution.

2. (Optional but highly recommended) Assay a small panel in one or two host like media. Ideally, we would like you to demonstrate that key rank order relationships (e.g., AncTF17–42 ≪ AncLFcin1 < AncLFcin2 < bLFcin; plus Q8R mutant effect) are robust across at least one more physiological environment, mitigating the “single medium in vitro” criticism. For this, you could choose 1–2 additional conditions that might be easiest to implement, including a synthetic nasal medium (SNM), a synthetic tear- or saliva-like medium, or a defined low-salt medium supplemented with physiologic ions. You would then test a small subset of peptides: hTF17–42, AncTF17–42, AncLFcin1, AncLFcin2, hLFcin, bLFcin, and (optionally) AncLFcin1_Q8R if you include experiment 1 (above). We do not require full CFU kinetics; a single end point viability readout at 24 h in the new medium will suffice. This will directly address Reviewer 1’s key conceptual critique regarding ecological relevance without requiring in vivo experiments. Even if absolute potency shifts, showing that relative potency and the effects of key substitutions are preserved will reassure reviewers that

your evolutionary conclusions are not an artifact of a single medium.

We have tested our series of peptides in two additional media conditions: 0.5% milk as well as OptiMEM media used for culturing of mammalian cells. Our general observations are recapitulated in these media conditions, i.e. lack of activity in hTF, AncTF, and reduced activity of ancestral peptides relative to bLFcin. There are some minor differences, such as a reduced activity of AncLFcin3 against *P. aeruginosa* in OptiMEM (Fig. S5). These minor discrepancies are perhaps not surprising given that changes in chemical/ionic environment are known to have substantial impacts on AMP effectiveness. We have included these data in revised Supplemental Figure 5, and include a discussion of the implications of these findings (~line 455).

Major points to address- Textual

1. Constrain evolutionary claims and integrate alternative scenarios.
The manuscript currently emphasizes a “stepwise origin” of antimicrobial function following lactoferrin gene duplication and suggests a largely gradual enrichment of cationic and hydrophobic residues in lactoferricin over time. Previous reviews and your own responses highlight that convergent substitutions, the presence of a distinct AMP in avian ovotransferrin, and the non monotonic trajectory of bactericidal activity argue for a more nuanced evolutionary picture.
Action: We ask that you (i) temper language implying strictly de novo mammal specific emergence, (ii) explicitly acknowledge the independent evolution of cryptic AMPs in related family members, and (iii) clearly distinguish between an overall trend in charge/hydrophobicity and the complex, non linear evolution of antimicrobial potency.

We have edited the language throughout the manuscript to address this concern – some of this was already incorporated into the previous manuscript before submission to *PLOS Biology*. We have also edited the title of the manuscript to remove the term “stepwise” in order to further address this issue. Additional discussion of other transferrin/lactoferrin-derived AMPs is also provided (~line 490).

2. Clarify timing relative to gene duplication.
Several statements imply that antimicrobial activity arose “after gene birth,” whereas your own data only resolve that AncTF17–42 lacks detectable activity and AncLFcin1 has modest activity.

Action: Please harmonize your terminology so that the Abstract, Results, and Discussion consistently discuss AMP emergence “around the time of gene duplication” and explicitly note the limited temporal resolution of ancestral reconstructions at that node.

We have edited the manuscript text throughout to address this point.

3. More fully acknowledge in vitro and medium specific limitations.
All functional assays are performed in a single low nutrient laboratory medium and on contemporary bacterial strains. Previous reviewers raised concerns about the ecological and physiological relevance of these conditions.
Action: While we do not require in vivo experiments, we do require an explicit, central discussion of these limitations: that AMP activity is highly context dependent, that ancestral bacterial strains cannot be recreated, and that your primary inferences concern relative differences among peptides and evolutionary tuning of host factors.

We have edited the manuscript text to address these points, particularly in the Discussion (~line 455).

4. Alternative functions of positively charged residues and trade offs.
The manuscript argues that increased positive charge facilitates targeting of negatively charged bacterial surfaces, but alternative functional explanations (e.g., iron binding properties, resistance to bacterial iron piracy, or changes in protease sensitivity) are plausible and were raised by prior reviewers.
Action: Please expand the Discussion to clearly articulate these alternative roles and to relate them to the observed non uniform trajectory of antimicrobial potency, while also clarifying that your hemolysis assays address only one potential toxicity trade off.

We have edited the Discussion to address these points (~lines 450).

5. Improve transparency of ancestral reconstruction and phylogeny.
Prior reviews requested more detail about the ancestral reconstruction procedure and phylogenetic context.
Action: We ask that you strengthen the Materials and Methods with explicit descriptions of taxon sampling, paralog discrimination, alignment treatment, and how your phylogeny relates to published lactoferrin trees. In the Results, please clearly explain the use of alternative (“Altall”) ancestral peptides and direct readers to the posterior probability plots and Newick file.

We have edited the manuscript to address these concerns (~lines 130, 560-575).

6. Clarify relationships between figures and experimental design.
There remains some confusion over the relationship between Fig. 2 and Fig. 3 and the nature of and statistical comparisons.
Action: Please revise the figure legends and relevant text to: (i) clearly describe that Fig. 2 presents normalized AUC heatmaps across peptide–bacteria–dose combinations; (ii) state that Fig. 3 displays independent experiments focusing on growth kinetics and CFU survival at selected doses; and (iii) fully describe normalization schemes and statistical thresholds.

We have edited the Figure 2 and 3 legends to address these concerns as well as modified text in the Methods (~line 600).

Minor points and additional editorial suggestions

We also encourage you to address the following points, which will improve clarity and accessibility:
• Reduce the use of the term “gene birth” and prefer “duplication,” “origin,” or “emergence of lactoferrin.”

We have removed the use of the term “gene birth” throughout the manuscript.

• Clarify that the enhanced activity conferred by Q5R has been experimentally demonstrated, whereas hypotheses about combinatorial cationic–aromatic pairs in bovine lactoferricin remain an open question.

We have removed hypotheses regarding mutation combinations from the Results section to address this point.

• Provide clearer statements about the relative susceptibility of Gram negative versus Gram positive bacteria to lactoferricin variants and avoid over interpreting differences as host specific adaptation without direct evidence.

We have removed speculation regarding host specific adaptation to lactoferrin and clarified differences between Gram-negative and positive susceptibility.

• Explicitly comment on the modest growth enhancement seen at low peptide

concentrations in some conditions and propose plausible mechanisms (e.g., nutrient use or resistant subpopulations).

We have added text to address this point (~lines 192).

• Ensure that all figure legends fully define color schemes, arrows/arrowheads, staining conditions, and statistical annotations, and that colors and shaded areas are easily visible.

We have added text to legends to help clarify figure labeling/colors.
