# Supplementary material for: Retracing the origin and evolution of a cryptic antimicrobial peptide within mammalian lactoferrin
Source: PLoS Biol. 2026 Aug 25;24(8):e3003932. doi: 10.1371/journal.pbio.3003932 (PMC13505947; doi:10.1371/journal.pbio.3003932)
Supplement: Supplementary file 3 [file pbio.3003932.s011.docx]

**Reconstructed ancestral sequences used for analysis**

Signal sequence trimmed from structural visualization

Equivalent region of human lactoferricin used in experiments

>AncTF(anc177)|avgPP:0.971|lnPP:-54.627|num_ambig:44|num_ambig_gaps:3

MRLAFPALLCCGALGLCLAAPEKTVRWCTISDHEASKCSSFRDNMKKADGPSVTCVKRTSYLECIKAIAANEADAVTLDGGLVFEAGLAPYNLKPVVAEVYGTEDDPQTHYYAVAVVKKGSNFQLNQLQGKKSCHTGLGRSAGWNIPIGTLLPLLNWPGPPEPLEKAVAKFFSGSCVPCADGKKFPNLCQLCAGKGTDKCACSSQEPYFGYSGAFKCLQDGAGDVAFVRHSTVFENLPNKADRDQYELLCPDNTRKPVDEYKECHLARVPSHAVVARSVDGKEDLIWELLNQAQEKFGKDKSAEFQLFGSPHGKDLLFKDSAHGFLRVPPKMDAGLYLGYEYITAIQNLREGEAPVAAESKKVKWCAVGHDERAKCDEWSAVSGGAIECASAETTEDCIAKIMKGEADAMSLDGGFIYIAGKCGLVPVLAENYKSKDSNGSDCVNRPVEGYYAVAVVKKSDADLTWNSLRGKKSCHTAVGRTAGWNIPMGLIYNQTRSCKFDEFFSQSCAPGSDPNSSLCALCIGSANPPNKCAPNSKERYYGYTGAFRCLVEKGDVAFVKHSTVLQNTDGKNPEAWAKDLKQEDFELLCLDGTRKPVTEAQSCHLARAPNHAVVSRKDKADFVRQVLLNQQAQFGRNGSECSGKFCLFQSETKDLLFKDNTECLAKLQDKTTYEKYLGPEYVTAVANLRQCSTSELLEACTFHRN

>AncTF_altAll|avgPP:0.966|lnPP:-72.809|num_ambig:44|num_ambig_gaps:3

MRLAFSALLSFGVLGLCLAVPEKTVRWCTVSDNEASKCSKFRENMKKADGPPVTCVKRTSYLECIKAIAANEADAVTLDGGLVYEAGLAPYNLKPVVAEFYGSEDEPQTHYYAVAVVKKGSDFQLNQLQGKKSCHTGLGRSAGWNIPIGTLLPFLNWPGPPEPLEKAVAKFFSGSCVPCADGKKFPNLCRLCAGKGTDKCACSSQEPYFGYSGAFKCLKDGAGDVAFVKHSTVFENLPNKADRDQYELLCPDNTRKPVDEYKDCHLARVPSHAVVARSVDGKEDLIWELLNQAQERFGKDKSAAFQLFGSPHGKDLLFKDSAHGFLRIPPKMDAGLYLGYEYFTAIQNLREGKAPVAAESKRVRWCAVGHDERAKCDEWSAVSGGKIECASAETTEDCIAKIMKGEADAMSLDGGFIYTAGKCGLVPVLAENYKSQDSNGSDCVNRPAEGYYAVAVVKKSDADLTWNSLKGKKSCHTAVGRTAGWNIPMGLIYNQTGSCKFDEFFSQSCAPGSDPNSNLCALCIGNSNPPNKCAPNSKERYYGYTGAFRCLVEKGDVAFVKHSTVLQNTDGKNPEAWAKDLKQEDFELLCLDGTRKPVTEAQNCHLARAPNHAVVSRKDKAEFVRQVLLNQQALFGRNGSDCPGMFCLFQSETKDLLFNDNTECLAKLQDKTTYEKYLGPEYVTAVANLRQCSTSELLEACTFHRS

>AncLF1(anc85)|avgPP:0.990|lnPP:-18.573|num_ambig:20|num_ambig_gaps:0

MRLVFPALLCLGALGLCLAAPRKTVRWCTISQDEAAKCSKFQQNMKKVGGPSVTCIKRTSYLECIQAIAANKADAVTLDGGLVFEAGLAPYKLRPVVAEVYGTEAEPQTHYYAVAVVKKGSNFQLNQLQGKKSCHTGLGRSAGWNIPIGTLRPFLNWTGPPEPLEKAVAKFFSASCVPCADGKQFPNLCRLCAGTGANKCACSSQEPYFGYSGAFKCLQDGAGEVAFVRDSTVFENLPNKADRDQYELLCPDNTRKPVDAFKECHLARVPSHAVVARSVNGKEDLIWELLRQAQEKFGKNKSPAFQLFGSPPGKKDLLFKDSALGFLRIPSKIDAGLYLGFSYFTAIQNLRETEAEVAARRKRVVWCAVGSEEQRKCEQWSSVSGGKVTCASAPTTEDCIALVMKGEADAMSLDGGFIYIAGKCGLVPVLAENQKSQESNNSDCVNRPVEGYLAVAVVRKSDADLTWNSLRGRKSCHTAVGRTAGWNIPMGLLFNQTGSCKFDEFFSQSCAPGSDPNSNLCALCIGDEQGQNKCAPNSKERYYGYTGAFRCLAEKAGDVAFVKDSTVLQNTDGKNTEAWAKDLKLEDFELLCLDGTRKPVTEAQSCHLAMAPNHAVVSRKDKAEHVEQVLLQQQAQFGRNGSDCPGKFCLFQSETKNLLFNDNTECLAKLQGKTTYEKYLGPEYVTAVANLRQCSTSPLLEACAFLRR

>AncLF1_altAll|avgPP:0.986|lnPP:-28.565|num_ambig:20|num_ambig_gaps:0

MRLVFPALLSLGALGLCLAAPRKTVRWCTISQDEAAKCSKFQENMKKVGGPPVTCVKRTSYLECIQAIAANKADAVTLDGGLVFEAGLAPYNLRPVVAEVYGTEAEPQTHYYAVAVVKKGSNFQLNQLQGKKSCHTGLGRSAGWNIPIGTLRPFLNWTGPPEPLEKAVAKFFSGSCVPCADGKQFPNLCQLCAGTGANKCACSSQEPYFGYSGAFKCLQDGAGDVAFVRDSTVFENLPNKADRDQYELLCPDNTRKPVDAFKECHLARVPSHAVVARSVNGKEDLIWELLRQAQEKFGKNKSPAFQLFGSPPGQKDLLFKDSAIGFLRIPSKIDAGLYLGFSYFTAIQNLRETEAEVAARRKRVVWCAVGSEEQRKCQQWSSVSGGKVTCASASTTEDCIALVMKGEADAMSLDGGFIYIAGKCGLVPVLAENQKSQESNNSDCVNRPVEGYLAVAVVRKSDADLTWNSLRGKKSCHTAVGRTAGWNIPMGLLFNQTGSCKFDEFFSQSCAPGSDPSSNLCALCIGDEKGQNKCAPNSKERYYGYTGAFRCLAEKAGDVAFVKDSTVLQNTDGKNTEAWAKDLKLEDFELLCLDGTRKPVTEAQSCHLAVAPNHAVVSRKDKAEYVEQVLLDQQAQFGRNGSDCPGKFCLFQSETKNLLFNDNTECLAKLQGKTTYEKYLGPEYVTAVANLRQCSTSPLLEACAFLSK

>AncLF2(anc73)|avgPP:0.991|lnPP:-15.736|num_ambig:23|num_ambig_gaps:0

MRLVFPALLFLGALGLCLAAPRRSVRWCTISQPEAAKCSKFQRNMKKVGGPSVSCIKKTSYLECIQAIAANKADAVTLDGGLVFEAGLAPYKLRPVAAEVYGTEAEPQTHYYAVAVVKKGSNFQLNQLQGVKSCHTGLGRSAGWNIPIGTLRPFLNWTGPPEPLEKAVAKFFSASCVPCADGKQFPNLCRLCAGTGANKCACSSQEPYFGYSGAFKCLQDGAGDVAFVRDSTVFENLPDKADRDQYELLCPDNTRKPVDAFKECHLARVPSHAVVARSVNGKEDLIWELLRQAQEKFGKNKSPAFQLFGSPPGQKDLLFKDSALGFLRIPSKIDAGLYLGFSYFTAIQNLRETEAEVAARRARVVWCAVGSEEQRKCEQWSSVSNGKVTCASASTTEDCIALVLKGEADAMSLDGGFIYIAGKCGLVPVLAENQKSQESNNSDCVNRPVEGYLAVAVVRKSDADITWNSLRGRKSCHTAVGRTAGWNIPMGLLFNQTGSCKFDEFFSQSCAPGSDPKSNLCALCIGDEQGENKCAPNSNERYYGYTGAFRCLAEKAGDVAFVKDVTVLQNTDGKNTEAWAKDLKLEDFELLCLDGTRKPVTEAQSCHLAMAPNHAVVSRKDKAEHLEQVLLDQQAQFGRNGSDCPGKFCLFQSETKNLLFNDNTECLAKLQGKTTYEKYLGPEYVTAIANLRQCSTSPLLEACAFLRR

>AncLF2_altAll|avgPP:0.987|lnPP:-28.289|num_ambig:23|num_ambig_gaps:0

MRLVFPALLFLGALGLCLAAPRKSVRWCTISQPEAAKCSKFQRNMKKVGGPPVSCVRRTSYLECIQAIAANKADAVTLDGGLVFEAGLDPYKLRPVAAEVYGTEAEPQTHYYAVAVVKKGSNFQLNQLQGVKSCHTGLGRSAGWNIPIGTLRPFLNWTGPPEPLEKAVAKFFSASCVPCADGKQYPNLCRLCAGTGANKCACSSQEPYFGYSGAFKCLQDGAGDVAFVRDSTVFENLPNKADRDQYELLCPDNTRKPVDAFKECHLARVPSHAVVARSVNGKEDLIWELLRQAQEKFGKNKSPAFQLFGSPPGQKDLLFKDSAIGFLRIPSKIDSGLYLGFNYFTAIQNLRETEAEVAARRARVVWCAVGSEEQRKCQQWSSVSGGKVTCASAPTTEDCIALVLKGEADAMSLDGGFIYIAGKCGLVPVLAENQKSQESNNPDCVNRPVEGYLAVAVVRKSDADLTWNSLRGKKSCHTAVGRTAGWNIPMGLLFNQTGSCKFDEFFSQSCAPGSDPRSNLCALCIGNEKGQNKCAPNSNERYYGYTGAFRCLAEKAGDVAFVKDVTVLQNTDGKNTEAWAKDLKLEDFELLCLDGTRKPVTEAQSCHLAMAPNHAVVSRKDKAEHLEQVLLQQQAQFGRNGSDCPGKFCLFQSETKNLLFNDNTECLAKLQGKTTYEKYLGPEYVTAIANLRQCSTSPLLEACAFLRK

>AncLF3(anc68)|avgPP:0.984|lnPP:-29.488|num_ambig:32|num_ambig_gaps:0

MKLFFPALLSLGALGLCLAAPRKSVRWCTISQPESSKCSQWQRRMKKVGGPSVTCVRKTSRLECIQAIAAKKADAVTLDGGLVFEAGLDPYKLRPVAAEVYGTEENPQTHYYAVAVVKKGSNFQLNQLQGLKSCHTGLGRSAGWNIPMGLLRPFLDWTGPPEPLQKAVAKFFSASCVPCADGKQYPNLCQLCAGTGENKCACSSQEPYFGYSGAFKCLQDGAGDVAFVKDSTVFENLPDKADRDQYELLCPDNTRKPVDAFKECHLARVPSHAVVARSVNGKEDLIWKLLSQAQEKFGKGKSSAFQLFGSPPGQKDLLFKDSALGFLRIPSKIDSGLYLGSNYLTAIQNLRETAAEVEARRARVVWCAVGPEEQRKCQQWSSQSNQNVTCATASTTEDCIALVLKGEADAMSLDGGYIYIAGKCGLVPVLAENQKSQESSSLDCVHRPVEGYLAVAVVRKANDGITWNSLRGKKSCHTAVDRTAGWNIPMGLLFNQTGSCKFDEFFSQSCAPGSDPKSNLCALCAGNEQGQNKCVPNSNERYYGYTGAFRCLAENAGDVAFVKDVTVLENTDGKNTEEWAKDLKLEDFELLCLDGTRKPVTEAESCHLAVAPNHAVVSRKDKAAHLEQVLLHQQAQFGRNGKDCPGKFCLFQSETKNLLFNDNTECLAKLQGKTTYEKYLGPEYVTAIANLRQCSTSPLLEACAFLMR

>AncLF3_altAll|avgPP:0.980|lnPP:-44.226|num_ambig:32|num_ambig_gaps:0

MKLFFPALLSLGALGLCLAAPRKSVRWCTISQPEASKCSQWQRRMKKVRGPSVSCIRKTSRLECIQAIAAKKADAVTLDGGLVFEAGLDPYKLRPVAAEVYGTEENPQTHYYAVAVVKKGSNFQLNQLQGVKSCHTGLGRSAGWNIPMGLLRPYLNWTGPPEPLQKAVAKFFSASCVPCVDGKEYPNLCQLCAGTGENKCACSSQEPYFGYSGAFKCLQDGAGDVAFVKDSTVFENLPEKADRDQYELLCPNNTRKPVDAFKECHLARVPSHAVVARSVNGKEDLIWKLLRKAQEKFGKGKPPAFQLFGSPPGQKDLLFKDSALGFLRIPSKIDSGLYLGSNYLTAIQGLRETAAEVEARRARVVWCAVGPEEQRKCQQWSSQSNQNVTCATASTTEDCIALVLKGEADAMSLDGGFIYIAGKCGLVPVLAENQKSPKSSNLDCVHRPVEGYLAVAVVRKANEDLTWNSLRGKKSCHTAVDRTAGWNIPMGLLFNQTGSCKFDEFFSQSCAPGSDPRSNLCALCVGNEEGQNKCVPNSNERYYGYTGAFRCLAENVGDVAFVKDVTVLDNTNGKNTEEWAKDLKLEDFELLCLDGTRKPVTEAQSCHLAVAPNHAVVSRKDKAAHLEQVLLHQQAQFGRNGQDCPGKFCLFQSETKNLLFNDNTECLAKLQGKTTYEKYLGSEYVTAIANLRQCSTSPLLEACAFLMR
