# Supplementary material for: Retracing the origin and evolution of a cryptic antimicrobial peptide within mammalian lactoferrin
Source: PLoS Biol. 2026 Aug 25;24(8):e3003932. doi: 10.1371/journal.pbio.3003932 (PMC13505947; doi:10.1371/journal.pbio.3003932)
Supplement: Peer review history

# Editor report: Decision Letter 0

**DOI:** 10.1371/journal.pbio.3003932.r001

**Authors:** Melissa Vazquez Hernandez

9 Apr 2026

Dear Dr Barber,

Thank you for submitting your manuscript entitled "Stepwise origin and evolution of a cryptic antimicrobial peptide in mammalian lactoferrin" for consideration as a Research Article by PLOS Biology.

Your manuscript has now been evaluated by the PLOS Biology editorial staff as well as by an academic editor with relevant expertise and I am writing to let you know that we would like to send your submission out for external peer review.

However, before we can send your manuscript to reviewers, we need you to complete your submission by providing the metadata that is required for full assessment. To this end, please login to Editorial Manager where you will find the paper in the 'Submissions Needing Revisions' folder on your homepage. Please click 'Revise Submission' from the Action Links and complete all additional questions in the submission questionnaire.

Once your full submission is complete, your paper will undergo a series of checks in preparation for peer review. After your manuscript has passed the checks it will be sent out for review. To provide the metadata for your submission, please Login to Editorial Manager (https://www.editorialmanager.com/pbiology) within two working days, i.e. by Apr 11 2026 11:59PM.

If your manuscript has been previously peer-reviewed at another journal, PLOS Biology is willing to work with those reviews in order to avoid re-starting the process. Submission of the previous reviews is entirely optional and our ability to use them effectively will depend on the willingness of the previous journal to confirm the content of the reports and share the reviewer identities. Please note that we reserve the right to invite additional reviewers if we consider that additional/independent reviewers are needed, although we aim to avoid this as far as possible. In our experience, working with previous reviews does save time.

If you would like us to consider previous reviewer reports, please edit your cover letter to let us know and include the name of the journal where the work was previously considered and the manuscript ID it was given. In addition, please upload a response to the reviews as a 'Prior Peer Review' file type, which should include the reports in full and a point-by-point reply detailing how you have or plan to address the reviewers' concerns.

During the process of completing your manuscript submission, you will be invited to opt-in to posting your pre-review manuscript as a bioRxiv preprint. Visit http://journals.plos.org/plosbiology/s/preprints for full details. If you consent to posting your current manuscript as a preprint, please upload a single Preprint PDF.

Feel free to email us at plosbiology@plos.org if you have any queries relating to your submission.

Kind regards,

Melissa

Melissa Vazquez Hernandez, Ph.D.

Associate Editor

PLOS Biology

mvazquezhernandez@plos.org

# Editor report: Decision Letter 1

**DOI:** 10.1371/journal.pbio.3003932.r002

**Authors:** Melissa Vazquez Hernandez

27 Apr 2026

Dear Matt,

Thank you for your patience while we considered your revised manuscript "Stepwise origin and evolution of a cryptic antimicrobial peptide in mammalian lactoferrin" for publication as a Research Article at PLOS Biology. Your revised study has been evaluated by the PLOS Biology editors and an Academic Editor with relevant expertise.

In light of the Academic Editor’s assessment of your revision and revision plan, which you will find in full below, we would like to invite you to revise your manuscript to address several key points raised during evaluation. The Academic Editor identified important areas where the work would benefit from additional experimental support and refinement of the framing. In particular, we ask you to directly test your central mechanistic hypothesis, that incremental changes in charge and hydrophobicity drive increased antimicrobial potency, through a minimal and targeted mutational analysis comparing AncLFcin1 and AncLFcin2, using the substitutions you previously proposed (or their reciprocal counterparts), and evaluating their effects using the same growth and CFU-based assays, with optional membrane permeabilization measurements. In addition, while not strictly required, we strongly encourage you to assess a small subset of peptides in one or two more physiologically relevant media to address concerns regarding ecological relevance and robustness of the observed activity patterns. We also think the manuscript would benefit from a more cautious and balanced presentation of the evolutionary narrative, including tempering claims of strictly stepwise or de novo emergence, clarifying the timing of functional changes relative to gene duplication, and explicitly acknowledging alternative evolutionary scenarios and functional interpretations. Please also clearly discuss the limitations of in vitro assays and medium-specific effects, expand consideration of alternative roles for positively charged residues and potential trade-offs, and improve transparency around ancestral reconstruction, phylogenetic context, and the use of alternative sequences. Finally, please revise figure legends and associated text to clarify experimental design, normalization, and statistical analyses, and address a number of minor points aimed at improving clarity and accessibility.

We believe you can make all these changes using textual changes and potentially some figure alterations, with only one (or potentially two) additional set of experiments. If you can satisfactorily address the major points above and incorporate the minor revisions where feasible, the manuscript will present a more balanced and rigorous account of the evolutionary history and functional diversification of lactoferricin. However, please keep in mind that we may still attempt to seek a second round of review from new reviewers, but the Academic Editor can decide based on your good-faith effort to thoroughly revise the paper based on these comments.

Given the extent of revision needed, we cannot make a decision about publication until we have seen the revised manuscript and your response to the reviewers' comments. Your revised manuscript is likely to be sent for further evaluation by all or a subset of the reviewers.

In addition to these revisions, you will need to complete some formatting changes, which you will receive in a follow up email. A member of our team will be in touch with a set of requests shortly.

We expect to receive your revised manuscript within 3 months. Please email us (plosbiology@plos.org) if you have any questions or concerns, or would like to request an extension.

At this stage, your manuscript remains formally under active consideration at our journal; please notify us by email if you do not intend to submit a revision so that we may withdraw it.

**IMPORTANT - SUBMITTING YOUR REVISION**

Your revisions should address the specific points made by each reviewer. Please submit the following files along with your revised manuscript:

1. A 'Response to Reviewers' file - this should detail your responses to the editorial requests, present a point-by-point response to all of the reviewers' comments, and indicate the changes made to the manuscript.

*NOTE: In your point-by-point response to the reviewers, please provide the full context of each review. Do not selectively quote paragraphs or sentences to reply to. The entire set of reviewer comments should be present in full and each specific point should be responded to individually, point by point.

You should also cite any additional relevant literature that has been published since the original submission and mention any additional citations in your response.

2. In addition to a clean copy of the manuscript, please also upload a 'track-changes' version of your manuscript that specifies the edits made. This should be uploaded as a "Revised Article with Changes Highlighted" file type.

*Re-submission Checklist*

When you are ready to resubmit your revised manuscript, please refer to this re-submission checklist: https://plos.io/Biology_Checklist

To submit a revised version of your manuscript, please go to https://www.editorialmanager.com/pbiology/ and log in as an Author. Click the link labelled 'Submissions Needing Revision' where you will find your submission record.

Please make sure to read the following important policies and guidelines while preparing your revision:

*Published Peer Review*

Please note while forming your response, if your article is accepted, you may have the opportunity to make the peer review history publicly available. The record will include editor decision letters (with reviews) and your responses to reviewer comments. If eligible, we will contact you to opt in or out. Please see here for more details:

https://blogs.plos.org/plos/2019/05/plos-journals-now-open-for-published-peer-review/

*PLOS Data Policy*

Please note that as a condition of publication PLOS' data policy (http://journals.plos.org/plosbiology/s/data-availability) requires that you make available all data used to draw the conclusions arrived at in your manuscript. If you have not already done so, you must include any data used in your manuscript either in appropriate repositories, within the body of the manuscript, or as supporting information (N.B. this includes any numerical values that were used to generate graphs, histograms etc.). For an example see here: http://www.plosbiology.org/article/info%3Adoi%2F10.1371%2Fjournal.pbio.1001908#s5

*Blot and Gel Data Policy*

We require the original, uncropped and minimally adjusted images supporting all blot and gel results reported in an article's figures or Supporting Information files. We will require these files before a manuscript can be accepted so please prepare them now, if you have not already uploaded them. Please carefully read our guidelines for how to prepare and upload this data: https://journals.plos.org/plosbiology/s/figures#loc-blot-and-gel-reporting-requirements

*Protocols deposition*

To enhance the reproducibility of your results, we recommend that if applicable you deposit your laboratory protocols in protocols.io, where a protocol can be assigned its own identifier (DOI) such that it can be cited independently in the future. Additionally, PLOS ONE offers an option for publishing peer-reviewed Lab Protocol articles, which describe protocols hosted on protocols.io. Read more information on sharing protocols at https://plos.org/protocols?utm_medium=editorial-email&utm_source=authorletters&utm_campaign=protocols

Thank you again for your submission to our journal. We hope that our editorial process has been constructive thus far, and we welcome your feedback at any time. Please don't hesitate to contact us if you have any questions or comments.

Sincerely,

Melissa

Melissa Vazquez Hernandez, Ph.D.

Associate Editor

PLOS Biology

mvazquezhernandez@plos.org

------------------------------------

COMMENTS FROM THE ACADEMIC EDITOR:

“Stepwise origin and evolution of a cryptic antimicrobial peptide in mammalian lactoferrin” by Sil et al.

The manuscript presents an integrative ancestral reconstruction and functional analysis of the lactoferricin domain of mammalian lactoferrin, documenting how cationic and hydrophobic features and antimicrobial potency have changed over deep time and on recent primate timescales. The work is clearly written, technically competent, and addresses questions of broad interest in protein evolution, innate immunity, and host–pathogen interactions. However, given the detailed prior peer review and the current framing, some revisions are required before further consideration.

Major points to address- Experimental

1. (Required) Test charge/hydrophobicity hypothesis with minimal mutant panel (AncLFcin1↔AncLFcin2) Directly connect the key substitutions between AncLFcin1 and AncLFcin2 to the observed jump in antimicrobial potency, strengthening the “stepwise tuning” narrative reviewers already engaged with. For this, we propose that you synthesize and test AncLFcin1_Q8R, AncLFcin1_T19S, and AncLFcin1_R23K (as you already proposed to PNAS). Alternatively, or in addition, you can synthesize and test the reciprocal mutations in AncLFcin2 (R8Q, S19T, K23R) to see if you can partially “revert” activity. You would be expected to carry out the same growth/AUC + CFU assays as Fig. 2–3 against one Gram negative (e.g., PAO1) and one Gram positive (e.g., S. aureus MN8). You could also assay the membrane permeabilization (PI uptake) at a single dose to see whether the major effect is on binding/permeabilization vs downstream killing. This will directly test your central mechanistic hypothesis (incremental gains in positive charge/hydrophobic packing drive increased antimicrobial activity), rather than inferring it from correlational ancestral reconstructions. We believe that this experiment provides a clean, minimalist “evolutionary swap” experiment that is reviewer friendly and squarely within your research protocols (short synthetic peptides in standard assays

2. (Optional but highly recommended) Assay a small panel in one or two host like media. Ideally, we would like you to demonstrate that key rank order relationships (e.g., AncTF17–42 ≪ AncLFcin1 < AncLFcin2 < bLFcin; plus Q8R mutant effect) are robust across at least one more physiological environment, mitigating the “single medium in vitro” criticism. For this, you could choose 1–2 additional conditions that might be easiest to implement, including a synthetic nasal medium (SNM), a synthetic tear- or saliva-like medium, or a defined low-salt medium supplemented with physiologic ions. You would then test a small subset of peptides: hTF17–42, AncTF17–42, AncLFcin1, AncLFcin2, hLFcin, bLFcin, and (optionally) AncLFcin1_Q8R if you include experiment 1 (above). We do not require full CFU kinetics; a single end point viability readout at 24 h in the new medium will suffice. This will directly address Reviewer 1’s key conceptual critique regarding ecological relevance without requiring in vivo experiments. Even if absolute potency shifts, showing that relative potency and the effects of key substitutions are preserved will reassure reviewers that your evolutionary conclusions are not an artifact of a single medium.

Major points to address- Textual

1. Constrain evolutionary claims and integrate alternative scenarios.

The manuscript currently emphasizes a “stepwise origin” of antimicrobial function following lactoferrin gene duplication and suggests a largely gradual enrichment of cationic and hydrophobic residues in lactoferricin over time. Previous reviews and your own responses highlight that convergent substitutions, the presence of a distinct AMP in avian ovotransferrin, and the non monotonic trajectory of bactericidal activity argue for a more nuanced evolutionary picture.

Action: We ask that you (i) temper language implying strictly de novo mammal specific emergence, (ii) explicitly acknowledge the independent evolution of cryptic AMPs in related family members, and (iii) clearly distinguish between an overall trend in charge/hydrophobicity and the complex, non linear evolution of antimicrobial potency.

2. Clarify timing relative to gene duplication.

Several statements imply that antimicrobial activity arose “after gene birth,” whereas your own data only resolve that AncTF17–42 lacks detectable activity and AncLFcin1 has modest activity.

Action: Please harmonize your terminology so that the Abstract, Results, and Discussion consistently discuss AMP emergence “around the time of gene duplication” and explicitly note the limited temporal resolution of ancestral reconstructions at that node.

3. More fully acknowledge in vitro and medium specific limitations.

All functional assays are performed in a single low nutrient laboratory medium and on contemporary bacterial strains. Previous reviewers raised concerns about the ecological and physiological relevance of these conditions.

Action: While we do not require in vivo experiments, we do require an explicit, central discussion of these limitations: that AMP activity is highly context dependent, that ancestral bacterial strains cannot be recreated, and that your primary inferences concern relative differences among peptides and evolutionary tuning of host factors.

4. Alternative functions of positively charged residues and trade offs.

The manuscript argues that increased positive charge facilitates targeting of negatively charged bacterial surfaces, but alternative functional explanations (e.g., iron binding properties, resistance to bacterial iron piracy, or changes in protease sensitivity) are plausible and were raised by prior reviewers.

Action: Please expand the Discussion to clearly articulate these alternative roles and to relate them to the observed non uniform trajectory of antimicrobial potency, while also clarifying that your hemolysis assays address only one potential toxicity trade off.

5. Improve transparency of ancestral reconstruction and phylogeny.

Prior reviews requested more detail about the ancestral reconstruction procedure and phylogenetic context.

Action: We ask that you strengthen the Materials and Methods with explicit descriptions of taxon sampling, paralog discrimination, alignment treatment, and how your phylogeny relates to published lactoferrin trees. In the Results, please clearly explain the use of alternative (“Altall”) ancestral peptides and direct readers to the posterior probability plots and Newick file.

6. Clarify relationships between figures and experimental design.

There remains some confusion over the relationship between Fig. 2 and Fig. 3 and the nature of normalization and statistical comparisons.

Action: Please revise the figure legends and relevant text to: (i) clearly describe that Fig. 2 presents normalized AUC heatmaps across peptide–bacteria–dose combinations; (ii) state that Fig. 3 displays independent experiments focusing on growth kinetics and CFU survival at selected doses; and (iii) fully describe normalization schemes and statistical thresholds.

Minor points and additional editorial suggestions

We also encourage you to address the following points, which will improve clarity and accessibility:

• Reduce the use of the term “gene birth” and prefer “duplication,” “origin,” or “emergence of lactoferrin.”

• Clarify that the enhanced activity conferred by Q5R has been experimentally demonstrated, whereas hypotheses about combinatorial cationic–aromatic pairs in bovine lactoferricin remain an open question.

• Provide clearer statements about the relative susceptibility of Gram negative versus Gram positive bacteria to lactoferricin variants and avoid over interpreting differences as host specific adaptation without direct evidence.

• Explicitly comment on the modest growth enhancement seen at low peptide concentrations in some conditions and propose plausible mechanisms (e.g., nutrient use or resistant subpopulations).

• Ensure that all figure legends fully define color schemes, arrows/arrowheads, staining conditions, and statistical annotations, and that colors and shaded areas are easily visible.

# Author comment: Author response to Decision Letter 2

**DOI:** 10.1371/journal.pbio.3003932.r003

24 Jun 2026

Attachment Submitted filename: Sil_PLOSBio_editorresponse_2026.pdf

# Editor report: Decision Letter 2

**DOI:** 10.1371/journal.pbio.3003932.r004

**Authors:** Melissa Vazquez Hernandez

13 Jul 2026

Dear Matt,

Thank you for your patience while we considered your revised manuscript "Origin and evolution of a cryptic antimicrobial peptide in mammalian lactoferrin" for publication as a Research Article at PLOS Biology. This revised version of your manuscript has been evaluated by the PLOS Biology editors and the Academic Editor.

Based on our Academic Editor's assessment of your revision, we are likely to accept this manuscript for publication, provided you satisfactorily address the following data and other policy-related requests.

1) We routinely suggest changes to titles to ensure maximum accessibility for a broad, non-specialist readership, and to ensure they reflect the contents of the paper. Please ensure you change both the manuscript file and the online submission system, as they need to match for final acceptance:

"Ancestral reconstruction reveals the origin and evolution of a cryptic antimicrobial peptide within mammalian lactoferrin"

2) Please add weblink of the funding agencies in the Financial Disclosure statement in the manuscript details.

3) Please add to your Competing Interests the declaration of pending patent application

4) You may be aware of the PLOS Data Policy, which requires that all data be made available without restriction: http://journals.plos.org/plosbiology/s/data-availability. For more information, please also see this editorial: http://dx.doi.org/10.1371/journal.pbio.1001797

Please supply the numerical values either in the a supplementary file or as a permanent DOI’d deposition for the following figures:

Figure 1C, 2A-F, 3A-F, 4ABD, 5B-E, 6B-G, S1B, S3A-D, S4, S5A-F, S6B-E, S7, S8

NOTE: the numerical data provided should include all replicates AND the way in which the plotted mean and errors were derived (it should not present only the mean/average values).

5) Please cite the location of the data clearly in all relevant main and supplementary Figure legends, e.g. “The data underlying this Figure can be found in S1 Data” or “The data underlying this Figure can be found in https://doi.org/10.5281/zenodo.XXXXX”

6) Please provide the tree files for the phylogenetic trees in Figures 1B, 2AB, S6A. Please make sure all relevant figures have scale bars.

7) Supplementary files (e.g., excel). Please ensure that all data files are uploaded as 'Supporting Information' and are invariably referred to (in the manuscript, figure legends, and the Description field when uploading your files) using the following format verbatim: S1 Data, S2 Data, etc. Multiple panels of a single or even several figures can be included as multiple sheets in one excel file that is saved using exactly the following convention: S1_Data.xlsx (using an underscore).

8) Please ensure that your Data Statement in the submission system accurately describes where your data can be found and is in final format, as it will be published as written there

9) Per journal policy, if you have generated any custom code during the course of this investigation, please make it available without restrictions. Please ensure that the code is sufficiently well documented and reusable, and that your Data Statement in the Editorial Manager submission system accurately describes where your code can be found. More information on our Code Policy, what and how to share can be found here: https://journals.plos.org/plosbiology/s/code-availability

Please note that we cannot accept sole deposition of code in GitHub, as this could be changed after publication. However, you can archive this version of your publicly available GitHub code to Zenodo. Once you do this, it will generate a DOI number, which you will need to provide in the Data Accessibility Statement (you are welcome to also provide the GitHub access information). See the process for doing this here: https://docs.github.com/en/repositories/archiving-a-github-repository/referencing-and-citing-content

As you address these items, please take this last chance to review your reference list to ensure that it is complete and correct. If you have cited papers that have been retracted, please include the rationale for doing so in the manuscript text, or remove these references and replace them with relevant current references. Any changes to the reference list should be mentioned in the cover letter that accompanies your revised manuscript.

In addition to these revisions, you may need to complete some formatting changes, which you will receive in a follow up email. A member of our team will be in touch with a set of requests shortly. If you do not receive a separate email within a few days, please assume that checks have been completed, and no additional changes are required.

We expect to receive your revised manuscript within two weeks.

To submit your revision, please go to https://www.editorialmanager.com/pbiology/ and log in as an Author. Click the link labelled 'Submissions Needing Revision' to find your submission record. Your revised submission must include the following:

- a cover letter that should detail your responses to any editorial requests, if applicable, and whether changes have been made to the reference list

- a Response to Reviewers file that provides a detailed response to the reviewers' comments (if applicable, if not applicable please do not delete your existing 'Response to Reviewers' file.)

- a track-changes file indicating any changes that you have made to the manuscript.

NOTE: If Supporting Information files are included with your article, note that these are not copyedited and will be published as they are submitted. Please ensure that these files are legible and of high quality (at least 300 dpi) in an easily accessible file format. For this reason, please be aware that any references listed in an SI file will not be indexed. For more information, see our Supporting Information guidelines:

https://journals.plos.org/plosbiology/s/supporting-information

*Published Peer Review History*

Please note that you may have the opportunity to make the peer review history publicly available. The record will include editor decision letters (with reviews) and your responses to reviewer comments. If eligible, we will contact you to opt in or out. Please see here for more details:

https://plos.org/published-peer-review-history/

*Press*

Should you, your institution's press office or the journal office choose to press release your paper, please ensure you have opted out of Early Article Posting on the submission form. We ask that you notify us as soon as possible if you or your institution is planning to press release the article.

*Protocols deposition*

To enhance the reproducibility of your results, we recommend that if applicable you deposit your laboratory protocols in protocols.io, where a protocol can be assigned its own identifier (DOI) such that it can be cited independently in the future. Additionally, PLOS ONE offers an option for publishing peer-reviewed Lab Protocol articles, which describe protocols hosted on protocols.io. Read more information on sharing protocols at https://plos.org/protocols?utm_medium=editorial-email&utm_source=authorletters&utm_campaign=protocols

Please do not hesitate to contact me should you have any questions.

Sincerely,

Melissa

Melissa Vazquez Hernandez, Ph.D.

Senior Editor

mvazquezhernandez@plos.org

PLOS Biology

# Author comment: Author response to Decision Letter 3

**DOI:** 10.1371/journal.pbio.3003932.r005

17 Jul 2026

Attachment Submitted filename: Sil_PLOSBio_editorresponse_2026_auresp_3.pdf

# Editor report: Decision Letter 3

**DOI:** 10.1371/journal.pbio.3003932.r006

**Authors:** Melissa Vazquez Hernandez

20 Jul 2026

Dear Matt,

Thank you for the submission of your revised Research Article "Retracing the origin and evolution of a cryptic antimicrobial peptide within mammalian lactoferrin" for publication in PLOS Biology. On behalf of my colleagues and the Academic Editor, Harmit S. Malik, I am pleased to say that we can in principle accept your manuscript for publication, provided you address any remaining formatting and reporting issues. These will be detailed in an email you should receive within 2-3 business days from our colleagues in the journal operations team; no action is required from you until then. Please note that we will not be able to formally accept your manuscript and schedule it for publication until you have completed any requested changes.

Please take a minute to log into Editorial Manager at http://www.editorialmanager.com/pbiology/, click the "Update My Information" link at the top of the page, and update your user information to ensure an efficient production process.

PRESS

We frequently collaborate with press offices. If your institution or institutions have a press office, please notify them about your upcoming paper at this point, to enable them to help maximise its impact. If the press office is planning to promote your findings, we would be grateful if they could coordinate with biologypress@plos.org. If you have previously opted in to the early version process, we ask that you notify us immediately of any press plans so that we may opt out on your behalf.

We also ask that you take this opportunity to read our Embargo Policy regarding the discussion, promotion and media coverage of work that is yet to be published by PLOS. As your manuscript is not yet published, it is bound by the conditions of our Embargo Policy. Please be aware that this policy is in place both to ensure that any press coverage of your article is fully substantiated and to provide a direct link between such coverage and the published work. For full details of our Embargo Policy, please visit http://www.plos.org/about/media-inquiries/embargo-policy/.

Thank you again for choosing PLOS Biology for publication and supporting Open Access publishing. We look forward to publishing your study.

Sincerely,

Melissa

Melissa Vazquez Hernandez, Ph.D.

Senior Editor

PLOS Biology

mvazquezhernandez@plos.org
